# Adiabatic state preparation of correlated wave functions with nonlinear scheduling functions and broken-symmetry wave functions

**DOI:** 10.1038/s42004-022-00701-8

**Published:** 2022-07-25

**Authors:** Kenji Sugisaki, Kazuo Toyota, Kazunobu Sato, Daisuke Shiomi, Takeji Takui

**Affiliations:** 1 Department of Chemistry and Molecular Materials Science, Graduate School of Science, Osaka City University, 3-3-138 Sugimoto, Sumiyoshi-ku, Osaka 558-8585 Japan; 2 JST PRESTO, 4-1-8 Honcho, Kawaguchi, Saitama 332-0012 Japan; 3 Centre for Quantum Engineering, Research and Education (CQuERE), TCG Centres for Research and Education in Science and Technology (TCG CREST), Sector V, Salt Lake, Kolkata, 700091 India; 4 Research Support Department/University Research Administrator Center, University Administration Division, Osaka City University, 3-3-138 Sugimoto, Sumiyoshi-ku, Osaka, 558-8585 Japan

**Keywords:** Quantum chemistry, Method development

## Abstract

Adiabatic state preparation (ASP) can generate the correlated wave function by simulating the time evolution of wave function under the time-dependent Hamiltonian that interpolates the Fock operator and the full electronic Hamiltonian. However, ASP is inherently unsuitable for studying strongly correlated systems, and furthermore practical computational conditions for ASP are unknown. In quest for the suitable computational conditions for practical applications of ASP, we performed numerical simulations of ASP in the potential energy curves of N_2_, BeH_2_, and in the *C*_2*v*_ quasi-reaction pathway of the Be atom insertion to the H_2_ molecule, examining the effect of nonlinear scheduling functions and the ASP with broken-symmetry wave functions with the **S**^2^ operator as the penalty term, contributing to practical applications of quantum computing to quantum chemistry. Eventually, computational guidelines to generate the correlated wave functions having the square overlap with the complete-active space self-consistent field wave function close to unity are discussed.

## Abstract (web summary)

Adiabatic state preparation (ASP) can generate correlated wave functions for quantum chemical calculations, but is inherently unsuitable for studying strongly correlated systems. Here, the authors perform numerical simulations of ASP for the ground state wave functions of molecules with strongly correlated electrons and propose practical conditions for preparation of close-to-exact correlated wave functions.

## Introduction

Quantum computing and quantum information processing (QC/QIP) is one of the most innovative research fields in the current central science^1^ and it has a potential to bring a paradigm shift in chemistry research. Among the diverse topics in the field of QC/QIP, sophisticated quantum chemical calculations have attracted attention as the near-future applications of quantum computers. Quantum chemical calculations are based on the Schrödinger equation that is a fundamental equation in quantum mechanics, and methods for accurate quantum chemical calculations potentially pave the way toward predictive quantum chemistry. Variationally best possible wave functions within the Hilbert space spanned by the basis set being used can be obtained by employing the full-configuration interaction (full-CI) calculations. However, the computational cost of full-CI methods scales exponentially against the number of basis functions relevant to the system size under study, and it easily reaches astronomical figures even for small molecules^2,3^.

To date, two major approaches for the quantum chemical calculations on quantum computers have been widely investigated, namely quantum phase estimation (QPE)-based full-CI calculations and a variational quantum eigensolver (VQE). The QPE-based approach was proposed in 2005^4^. It is based on the quantum simulations of the time evolution of an approximated wave function and projective measurement to the eigenstate of a given Hamiltonian. The quantum circuit for the QPE-based full-CI is too deep to be executed on noisy intermediate-scale quantum (NISQ) devices currently available. The QPE-based approach, however, is expected to be a powerful tool when fault-tolerant quantum computing (FTQC) becomes available, because the computational cost of the QPE-based full-CI scales polynomially against the system size, and therefore exponential improvement of the computational cost scaling from the classical counterpart is guaranteed theoretically. In the QPE-based methods the time evolution of wave functions should be simulated conditionally on the ancillary qubit, but this requirement is recently removed by the appearance of the approach based on the Bayesian phase difference estimation (BPDE) algorithm^5^. VQE is a quantum–classical hybrid algorithm, and it utilizes parametrized quantum circuits to prepare correlated wave functions and computes energy expectation values by statistical sampling of the measurement outcome^6^. Classical computers are used to perform the variational optimizations of parameters relevant to the quantum circuits. VQE has been extensively studied from both the experimental and theoretical sides, because the parametrized quantum circuit used for wave function preparation is usually very shallow enough to be executed on NISQ devices. It should be noted that, however, the computational cost scaling of the VQE-based approaches has not been sorely elucidated yet, and it is unclear whether quantum chemical calculations can be accelerated by using VQE. For example, VQE calculations with the unitary coupled cluster with singles and doubles (UCCSD) ansatz scale polynomial. The approximate UCCSD calculations, however, can also be done with the polynomial cost on a classical computer, by solving the amplitude equation of the similarity transformed Hamiltonian. Thus, the computational cost scaling becomes polynomial vs. polynomial for the UCCSD calculations on quantum and classical computers. Also, because the full-CI wave function contains an exponentially large number of variables, solving the full-CI using VQE with naïve implementation scales exponential. Recent reviews on quantum chemical calculations on quantum computers including QPE and VQE can be found elsewhere^7–13^.

In the QPE-based full-CI calculations, the preparation of a “good” approximated wave function having sufficiently large overlap with the exact wave function of the target electronic state is crucial. This is because the probability of which eigenenergy of the electronic state can be obtained in the QPE is proportional to the square overlap between the approximated and the exact wave functions. If the approximated wave function has exponentially small overlap with the eigenfunction of the target electronic state, an exponentially large number of QPE experiments are required to acquire the correct results, which spoils the advantages of a quantum speedup. In the equilibrium geometry of typical closed-shell singlet molecules and open-shell high-spin molecules carrying no unpaired electrons of spin-β, the Hartree–Fock (HF) wave function |Ψ_HF_〉 dominantly contributes to the full-CI wave function of the electronic ground state, and the use of |Ψ_HF_〉 is generally a good choice. In the low-spin states of open-shell molecules, by contrast, the wave function is approximated by the linear combination of several Slater determinants so as to be an eigenfunction of the **S**^2^ operator, and the overlap between |Ψ_HF_〉 and |Ψ_full-CI_〉 becomes small. We demonstrated that the symmetry-adapted configuration state function (CSF) can have a large overlap with the full-CI wave function of open-shell low-spin systems, and proposed quantum circuits to prepare the |Ψ_CSF_〉 on a quantum computer^14,15^. We also reported an approach to generate multi-configurational wave functions on quantum computers without performing any post-HF calculations^16^, which is suitable for the study of the systems with intermediate open-shell characters like molecules under covalent bond dissociations. These approaches can effectively consider static (nondynamical) electron correlation effects.

Although these approaches are useful to treat electronic states of open-shell molecules, the overlap with the full-CI wave function becomes small when dynamical electron correlation effects are also significant. Molecules having electronic structures too complicated to deal with facile approaches like density functional theory (DFT) are naturally one of the main targets of sophisticated quantum chemical calculations, and the development of theoretical methods to generate correlated wave functions considering both static and dynamical electron correlation effects on quantum computers is an important task. Note that one of the anticipated usage of VQE is the preparation of approximate wave functions used as the input for QPE^17^, but recent numerical simulations of VQE of the *C*_2*v*_ quasi-reaction pathway of Be + H_2_ → BeH_2_ reaction revealed that the variational parameter optimization converges very slowly for strongly correlated systems^18^.

Noticeably, recent years have witnessed that these promising approaches are relevant to innovative development in simulating quantum systems, such as Hamiltonian simulation. Adiabatic state preparation (ASP)^4^ is an approach to generate correlated wave functions based on an adiabatic theorem^19^. ASP belongs to an adiabatic quantum algorithm^20,21^, in which the wave function of the ground state of a problem Hamiltonian *H*_*P*_ is generated adiabatically, by starting from the ground state wave function of an initial Hamiltonian *H*_*I*_ and slowly varying the Hamiltonian by using a scheduling function *s*(*t*) in Eq. 1 from 0 to 1.

1 $$H\left(t\right)=\left(1-s\left(t\right)\right){H}_{I}+s\left(t\right){H}_{P}$$

The scheduling function is often set as *s*(*t*) = *t*/*T*, where *T* is a total evolution time length. By using |Ψ_HF_〉 and a Fock operator as the initial wave function and Hamiltonian, respectively, and the full Hamiltonian as *H*_*P*_, we can obtain |Ψ_full-CI_〉 if the evolution time *T* is long enough^4^. Note that an approach using the maximum commuting Hamiltonian as the initial Hamiltonian *H*_*I*_ was proposed recently^22^. ASP was adopted for the QPE-based full-CI of H_2_ molecule using an NMR quantum computer^23^. ASP numerical simulations of the 1 ^1^A_1_ state of methylene (CH_2_) molecule was reported in 2014^24^, and the optimization of the scheduling procedure by using VQE was discussed in 2021^25^. Application of the complete active space configuration interaction (CASCI) wave function as the initial wave function and the nonlinear scheduling functions *s*(*t*) = (*t*/*T*)^*c*^ (0 < *c* < 1) was also reported in 2021^26^.

Although ASP is a long-term (or FCTC) algorithm because the quantum circuit for ASP is usually too deep to execute on NISQ devices, it is promising because it does not require detailed a priori knowledge of the electronic structure of the system being studied. However, ASP is potentially unsuitable for strongly correlated systems, because the evolution time length *T* should be set longer when it is applied to the systems with smaller energy gaps between the ground and excited states. We also emphasize that the number of ASP studies so far documented are very few, and there has been little knowledge on the optimal computational conditions of ASP, such as the setting of the evolution time length and the selection of scheduling functions. Then, it is essential to explore suitable computational conditions which help make ASP as a practical tool for the preparation of correlated wave functions, which is the main subject of this work. Note that variational quantum imaginary time evolution (QITE) is also available for the preparation of correlated wave functions^27–30^. However, the total length of imaginary time propagation is determined by the spectrum of the Hamiltonian and the initial overlap with the exact wave function, and the application of QITE to strongly correlated systems is also an important issue.

**Fig. 1:**
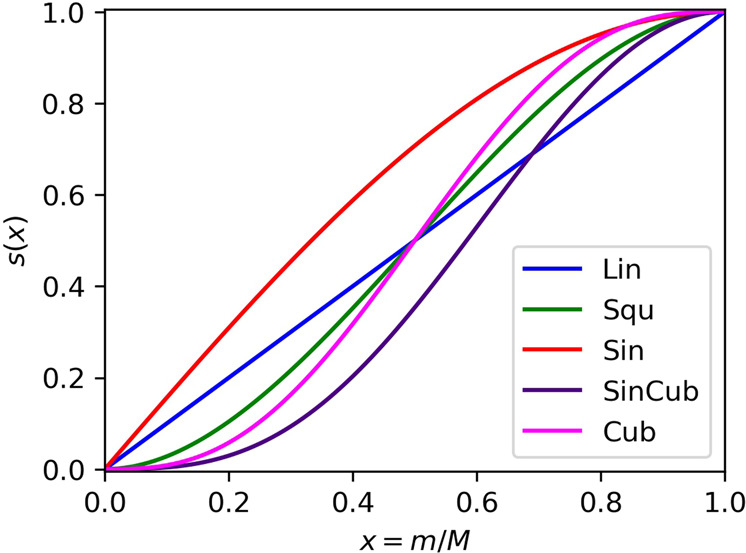
Plots of the scheduling functions tested in Eq. 1. Mathematical definitions of the scheduling functions are provided in Table 1.

In this work, we have carried out numerical simulations of ASP for the generation of correlated wave functions in the triple bond dissociation of N_2_ molecule, the symmetric bond dissociation of BeH_2_ molecule, and the *C*_2*v*_ quasi-reaction pathway of beryllium atom insertion to H_2_ molecule. We have explored the effects of nonlinear scheduling functions and the ASP starting with a broken-symmetry (BS) wave function |Ψ_BS_〉 by using the electron spin **S**^2^ operator as the penalty term in the time-dependent Hamiltonian. As the scheduling function *s*(*t*) in Eq. 1, we examined five different functions listed in Table 1 and plotted in Fig. 1; all of them were studied as the scheduling function in adiabatic algorithms by Hu and Wu^31^. An anticipated application of ASP is the wave function preparation for QPE, and thus in this study the quality of the wave functions obtained from ASP is evaluated by means of the square overlap with the CASCI wave function, |〈Ψ_ASP_|Ψ_CASCI_〉|^2^. Note that to use ASP for the wave function preparation in QPE, the evolution time required for ASP must be significantly shorter than that needed in QPE (*T* ~ 2000 atomic unit to achieve 1 kcal mol^−1^ of energy precision^16^). Eventually, we attempt to propose guidelines for the evolution time length and the selection for starting wave functions toward the practical use of ASP.

**Table 1:** Scheduling functions *s*(*x*) (*x* = *m*/*M*) tested in Eq. 1.

Name and abbreviation	Function
Linear (Lin)	$s\left(x\right)=x$
Square (Squ)	$s\left(x\right)=3{x}^{2}-2{x}^{3}$
Sinusoidal (Sin)	$s\left(x\right)={{\sin }}\left(\pi x/2\right)$
Sinusoidal cubic (SinCub)	$s\left(x\right)={{{\sin }}}^{3}\left(\pi x/2\right)$
Cubic (Cub)	$s\left(x\right)=6{x}^{5}-15{x}^{4}+10{x}^{3}$

## Results and discussion

### Scheduling function and evolution time length dependences of ASP in the potential energy curve of N_2_

**Fig. 2:**
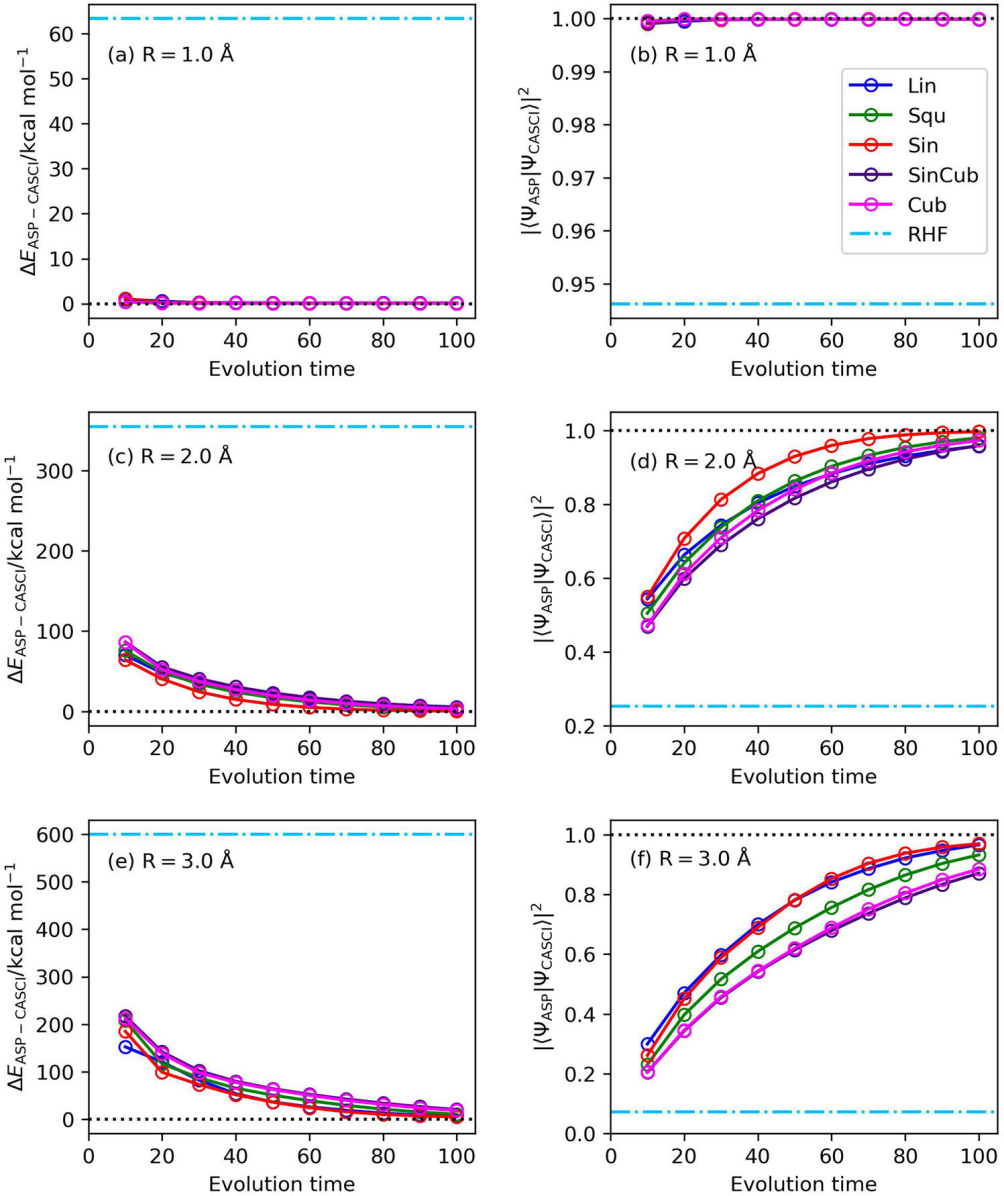
Results of the numerical quantum circuit simulation of N_2_ molecule. **a**,**c**,**e** The energy deviations from the CASCI values in (**a**) R(N–N) = 1.0 Å, (**c**) R(N–N) = 2.0 Å, and (**e**) R(N–N) = 3.0 Å. **b**, **d**, **f** The square overlaps with the CASCI wave function in (**b**) R(N–N) = 1.0 Å, (**d**) R(N–N) = 2.0 Å, and (**f**) R(N–N) = 3.0 Å.

**Fig. 3:**
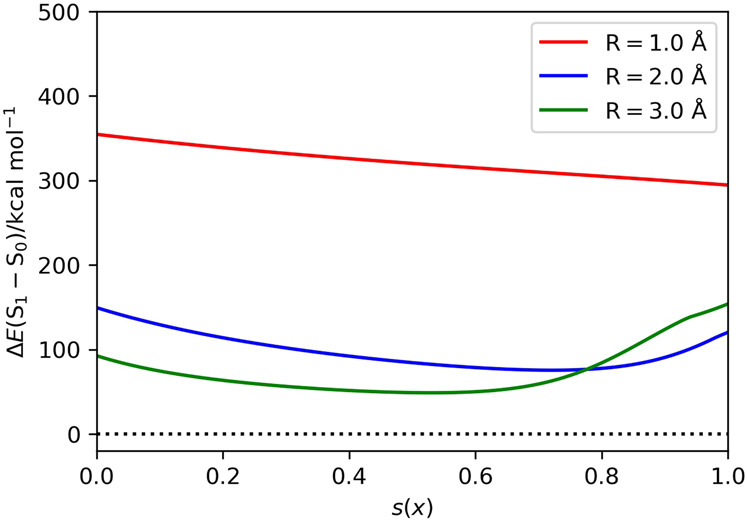
The S_1_−S_0_ energy gap of the instantaneous Hamiltonian *H*(*s*(*x*)) of N_2_ molecule with the bond lengths R(N−N) = 1.0, 2.0, and 3.0 Å. The Δ*E*(S_1_ − S_0_) values at *s*(*x*) = 0.0 and 1.0 corresponds to the energy gap of the Fock operator and the full electronic Hamiltonian, respectively.

First, we explored the scheduling function and the evolution time length dependences of ASP in N_2_ molecule with R(N–N) = 1.0, 2.0, and 3.0 Å. These geometries are selected as the representatives of the structures close to the equilibrium geometry where |〈Ψ_HF_|Ψ_CASCI_〉|^2^ is large, in the intermediate bond dissociation region where neither the |Ψ_HF_〉 nor the |Ψ_CSF_〉 are good approximation of the ground state, and in the bond dissociated region where the |Ψ_CASCI_〉 can be approximate to a |Ψ_CSF_〉. The numerical quantum circuit simulation results with *T* = 10–100 are summarized in Fig. 2. The number of quantum gates required for a single Trotter step is about 6900. At the geometry R(N–N) = 1.0 Å, ASP gives the wave function close to the |Ψ_CASCI_〉 even for the shortest evolution time length being tested (*T* = 10) regardless of the choice of the scheduling functions. The scheduling function and evolution time length dependences become significant for the longer N–N distances. Among the five scheduling functions being tested, the sinusoidal function exhibits the fastest convergence to the CASCI wave function against the evolution time length *T*. By employing the sinusoidal function as the scheduling function, we need *T* = 50 and 70 for R(N–N) = 2.0 and 3.0 Å, respectively, to achieve |〈Ψ_ASP_|Ψ_CASCI_〉|^2^ > 0.9. Longer evolution time is required to achieve the same magnitude of the square overlap for the elongation of the N–N bond, because the HOMO–LUMO gap and the energy gap between the ground and excited states become smaller for the elongated N–N distances. Plots of the S_1_−S_0_ energy gap of the instantaneous Hamiltonian (Fig. 3) indicate that the *s*(*x*) value giving the minimum Δ*E*(S_1_−S_0_) value becomes larger for the shorter R(N−N) values.

**Fig. 4:**
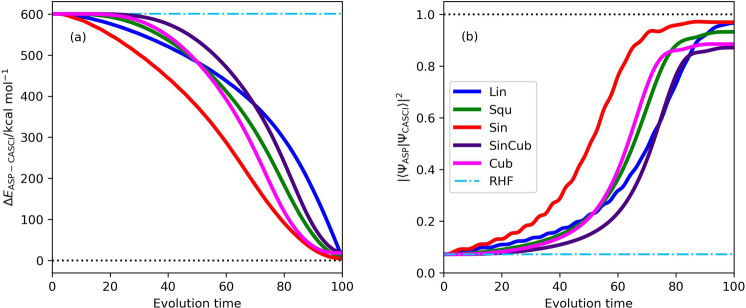
Trajectories of ASP with *T* = 100 in N_2_ molecule with R(N–N) = 3.0 Å. **a** The energy deviations from the CASCI value. **b** The square overlaps with the CASCI wave function.

The trajectories of ASP of N_2_ in the geometry R(N–N) = 3.0 Å with the evolution time length *T* = 100 are plotted in Fig. 4. Among the five scheduling functions, the sinusoidal function gives the smallest Δ*E*_ASP−CASCI_ value and the largest square overlap at each time step. Note that the trajectories of the square overlap calculated by using the linear and sinusoidal functions oscillate in the beginning of ASP. This originates from the fact that the gradient ∂*E*(*t*)/∂*s*(*t*) ≠ 0 at *t* = 0^31^. The same trends were also observed in the other geometries and molecules under study.

The reason why the sinusoidal function gave the best results among the five scheduling functions can be explained by the structure of the HF wave function and the energy landscape of the instantaneous Hamiltonian. One has to set the evolution time length prior to computation to apply ASP. The evolution time length *T* is usually set to satisfy the condition in Eq. 2^32^.

2 $$\mathop{{{\max }}}\limits_{s\in \left[0,1\right]}\frac{\left|\left\langle {\Psi }_{g}\left(s\right)|{\partial }_{s}H\left(s\right)|{\Psi }_{e}\left(s\right)\right\rangle \right|}{{\left|{\varepsilon }_{e}\left(s\right)-{\varepsilon }_{g}\left(s\right)\right|}^{2}}\ll T$$

Here, |Ψ_*g*_(*s*)〉 and |Ψ_*e*_(*s*)〉 are the wave functions of the electronic ground and excited states, respectively, of the instantaneous Hamiltonian *H*(*s*), and *ε*_*g*_(*s*) and *ε*_*e*_(*s*) are the corresponding energy eigenvalues. From Eq. 2, we can sweep the time-dependent Hamiltonian faster if the energy gaps between the ground and the excited states are large and if the numerator of Eq. (2) is small. The |Ψ_HF_〉 can be rewritten as |Ψ_HF_〉 = Σ_*j*_ *c*_*j*_|Ψ_*j*_〉, where |Ψ_*j*_〉 is the *j*th eigenfunction and *c*_*j*_ is the corresponding coefficient. Because the |Ψ_HF_〉 is spin and spatial symmetry-adapted, only the excited states belonging to the same spin and spatial symmetry can have non-negligible contribution to the numerator of Eq. 2 in the absence of Trotter decomposition errors and noises. The excitation energies of the corresponding excited states in the weakly correlated regime are generally larger in the earlier stage of ASP. This is because the Fock operator used as the initial Hamiltonian contains the terms with occupied orbitals only, and the excited states cannot be sufficiently stabilized under the Fock operator. Thus, we can sweep the Hamiltonian faster in the earlier stage of ASP, and the sweep speed must be attenuated by the time evolution. The sinusoidal function has such a structure.

Note that Eq. 2 is insufficient, and it does not guarantee the validity of the adiabatic approximation^33,34^. In fact, Marzlin and Sanders claimed that the application of the adiabatic theorem may lead to an inconsistency no matter how slowly the Hamiltonian is varied^33^. They also pointed out that the inconsistency becomes a potential problem whenever |Ψ(*T*)〉 deviates greatly from the initial state |Ψ(0)〉. Strongly correlated systems can be the case of such a small overlap |〈Ψ(0)|Ψ(*T*)〉| if the |Ψ_HF_〉 is employed as |Ψ(0)〉, and therefore the application of ASP to strongly correlated systems is a challenging problem. The necessary and sufficient condition for adiabatic evolution was discussed by Wang and Plenio, by decomposing the diabatic propagator into the geometric functions determined by the eigenstates and the modulation functions determined by the energy gaps and the speed of sweeping^35^. By utilizing these necessary and sufficient conditions for adiabatic condition, Xu and coworkers demonstrated the adiabatic evolution in the presence of vanishing energy gaps, using a nitrogen-vacancy center in diamond^36^. It should be also noted that approaches based on the shortcuts to adiabaticity (STA) have been eagerly studied to manipulate the quantum system on timescales shorter than decoherence time^37^, but it requires some non-physical Hamiltonian in order to make it work, and it is still an open and challenging problem.

**Fig. 5:**
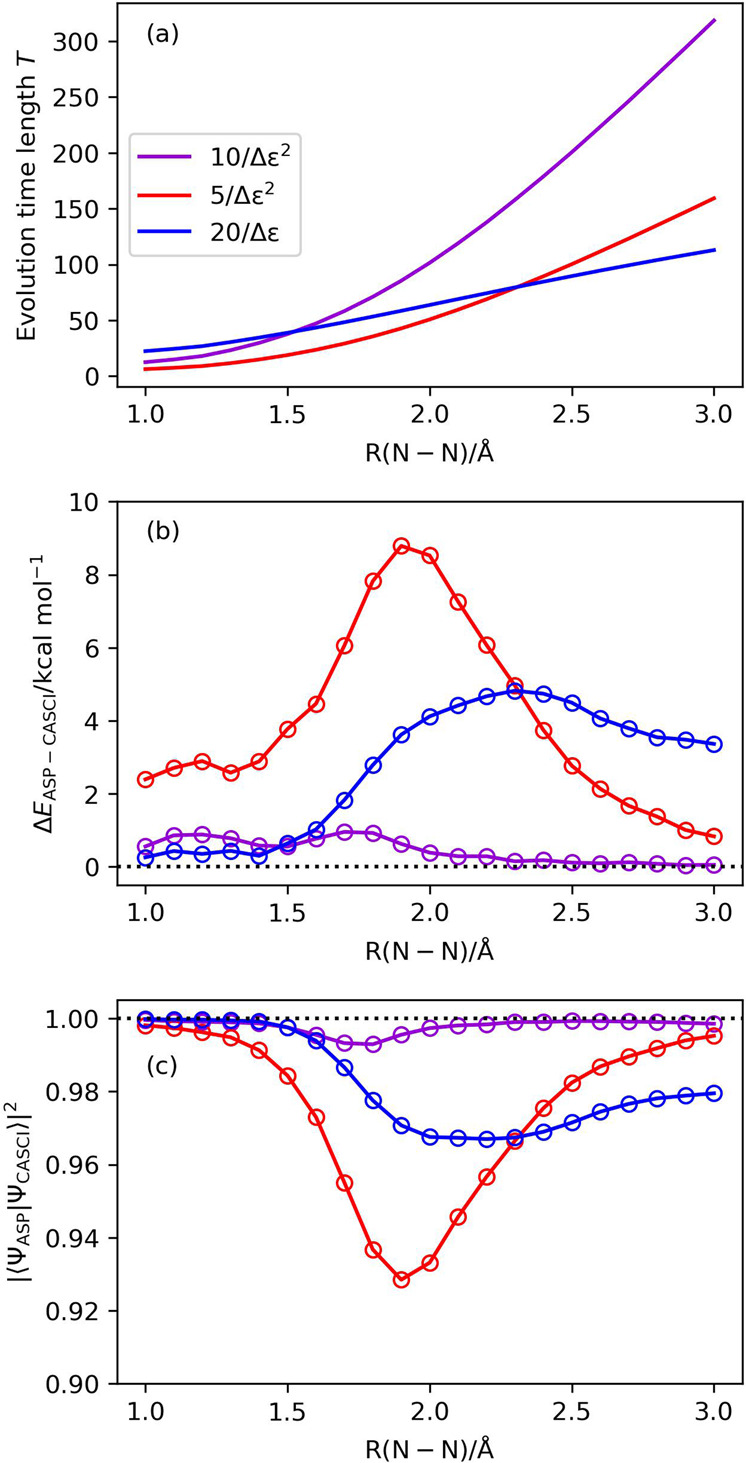
Results of the numerical simulation of ASP with different evolution time length determination strategies in N_2_. Sinusoidal function is used for the scheduling function. **a** Evolution time length being tested. **b** The energy deviations from the CASCI values. **c** The square overlaps with the CASCI wave functions.

Although Eq. 2 may lead to an inconsistency of the adiabatic evolution^33,34^, it can be used as the guideline of the evolution time length. Indeed, the variation of the wave function under the time-dependent Hamiltonian, which is responsible for the inconsistency, may be difficult to estimate in advance of the ASP simulation, but the energy gap between the ground and the first excited states can be roughly evaluated from the HOMO–LUMO gap Δ*ε* = *ε*(LUMO) − *ε*(HOMO), where *ε*(HOMO) and *ε*(LUMO) are the orbital energies of HOMO and LUMO, respectively. We note that the HOMO–LUMO gap estimation is only a practical approach. The exact energy landscape is required to fully adjust the scheduling function *s*(*x*), which corresponds to solving the problem altogether. However, the HOMO–LUMO gap can be used for crude estimation of the excitation energy. These orbital energies can be obtained from the HF calculations, and therefore using the HOMO–LUMO gap for the determination of the evolution time length does not need additional computation. Excitation energy estimations based on the single excitation CI or time-dependent DFT may be more reliable, but such computations inherently raise the computational cost. Based on this strategy, we examined the following evolution time determination methods: *T* = 5/Δ*ε*^2^ and *T* = 10/Δ*ε*^2^. In addition to them, we also tested the evolution time length *T* = 20/Δ*ε*. The results of the quantum circuit simulations with different evolution time length determination strategies with the sinusoidal scheduling function are summarized in Fig. 5. If we adopt the strategy *T* = 10/Δ*ε*^2^, we can obtain the correlated wave function with the square overlap |〈Ψ_ASP_|Ψ_CASCI_〉|^2^ > 0.99 at all bond lengths under study, but the evolution time for the longer N–N distance is quite long. The strategy *T* = 5/Δ*ε*^2^ gives fairly good results except for intermediate bond dissociation region. Considering the evolution time lengths and the square overlap, the strategy *T* = 20/Δ*ε* seems to be most suitable for ASP of the triple bond dissociation in N_2_. The same trend was observed for the other scheduling functions, although the deviations from the CASCI result are larger for the other functions than the sinusoidal one (see Supplementary Fig. S2).

### ASP with broken-symmetry wave functions and the S^2^ penalty term in the potential energy curve of N_2_

It is naturally expected that the use of multiconfigurational wave functions gives a plausible choice for the starting wave function in strongly correlated systems. In fact, Kremenetski and coworkers reported that substantial speedup of ASP can be achieved by using the CASCI wave function as the starting wave function^26^. However, encoding the CASCI wave function on a quantum register becomes nontrivial when the active space of CASCI is large. Here, we examine an alternative and facile approach of ASP for strongly correlated systems by adopting the BS wave function |Ψ_BS_〉 as the starting wave function with the **S**^2^ operator as the penalty term in the time-dependent Hamiltonian. The BS methods have been widely used in the DFT calculations of open-shell low-spin states such as spin-singlet states of biradicals^38–40^. The |Ψ_BS_〉 is a single Slater determinant with spin-β unpaired electrons in the localized singly occupied molecular orbitals (SOMOs). The |Ψ_BS_〉 is an eigenfunction of the **S**_*z*_ operator but is not an eigenfunction of the **S**^2^ operator, and thus the |Ψ_BS_〉 is expressed by a linear combination of wave functions having different spin quantum numbers *S*, as in Eqs. 3 and 4 for four- and six-spin *M*_*S*_ = 0 BS wave functions, respectively, for example.

3 $$|\alpha \alpha \beta \beta \rangle =\frac{1}{\sqrt{6}}|{\Psi }_{S=2,{M}_{S}=0}\rangle +\frac{1}{\sqrt{2}}|{\Psi }_{S=1,{M}_{S}=0}\rangle +\frac{1}{\sqrt{3}}|{\Psi }_{S=0,{M}_{S}=0}\rangle$$

4 $$|\alpha \alpha \alpha \beta \beta \beta \rangle =	\, \frac{1}{\sqrt{20}}|{\Psi }_{S=3,{M}_{S}=0}\rangle +\frac{1}{2}|{\Psi }_{S=2,{M}_{S}=0}\rangle +\frac{3}{\sqrt{20}}|{\Psi }_{S=1,{M}_{S}=0}\rangle \\ 	+\frac{1}{2}|{\Psi }_{S=0,{M}_{S}=0}\rangle$$

The coefficients in the right-hand side of Eqs. 3 and 4 can be derived from the structure of spin eigenfunctions^41^. The eigenvalue of the **S**^2^ operator is *S*(*S* + 1), and the expectation values of the **S**^2^ operator of the wave functions given in Eqs. 3 and 4 are calculated to be 2.0 and 3.0, respectively.

Because the quantum state corresponding to the |Ψ_BS_〉 can be prepared on a quantum computer with the same cost as the preparation of |Ψ_HF_〉 regardless of the number of singly occupied orbitals, it is possible to use the |Ψ_BS_〉 as the starting wave function in ASP. The wave function components having the spin quantum number different from the target electronic state can be eliminated by introducing the **S**^2^ operator as the penalty term in the time-dependent Hamiltonian *H*(*t*), as in Eq. 5.

5 $$H\left(t\right)=\left(1-s\left(t\right)\right){H}_{I}+s\left(t\right){H}_{P}+s\left(t\right)c{{{{{{\bf{S}}}}}}}^{2}$$

Here, *c* is a coefficient that controls the strength of the penalty term. The **S**^2^ operator as the penalty term works to raise the energy of the wave function with the spin quantum number *S* by *cS*(*S* + 1), and therefore spin contaminants can be readily eliminated during ASP. We expect that ASP starting from |Ψ_BS_〉 can generate multiconfigurational wave functions efficiently, just as the spin-projected extended Hartree–Fock (EHF) method^42^ in classical computation that applies the spin projection operator to the spin contaminated UHF wave function. In Eq. 3, larger *c* values can shift the energies of the spin contaminants greatly, but too large *c* values will result in departure from the adiabatic pathway. Note that we have already proposed a method to construct a quantum circuit for the time evolution operator exp(−*is*(*t*)*c***S**^2^*t*) by utilizing a generalized spin coordinate mapping^43^, which can be directly used for the present ASP study.

**Fig. 6:**
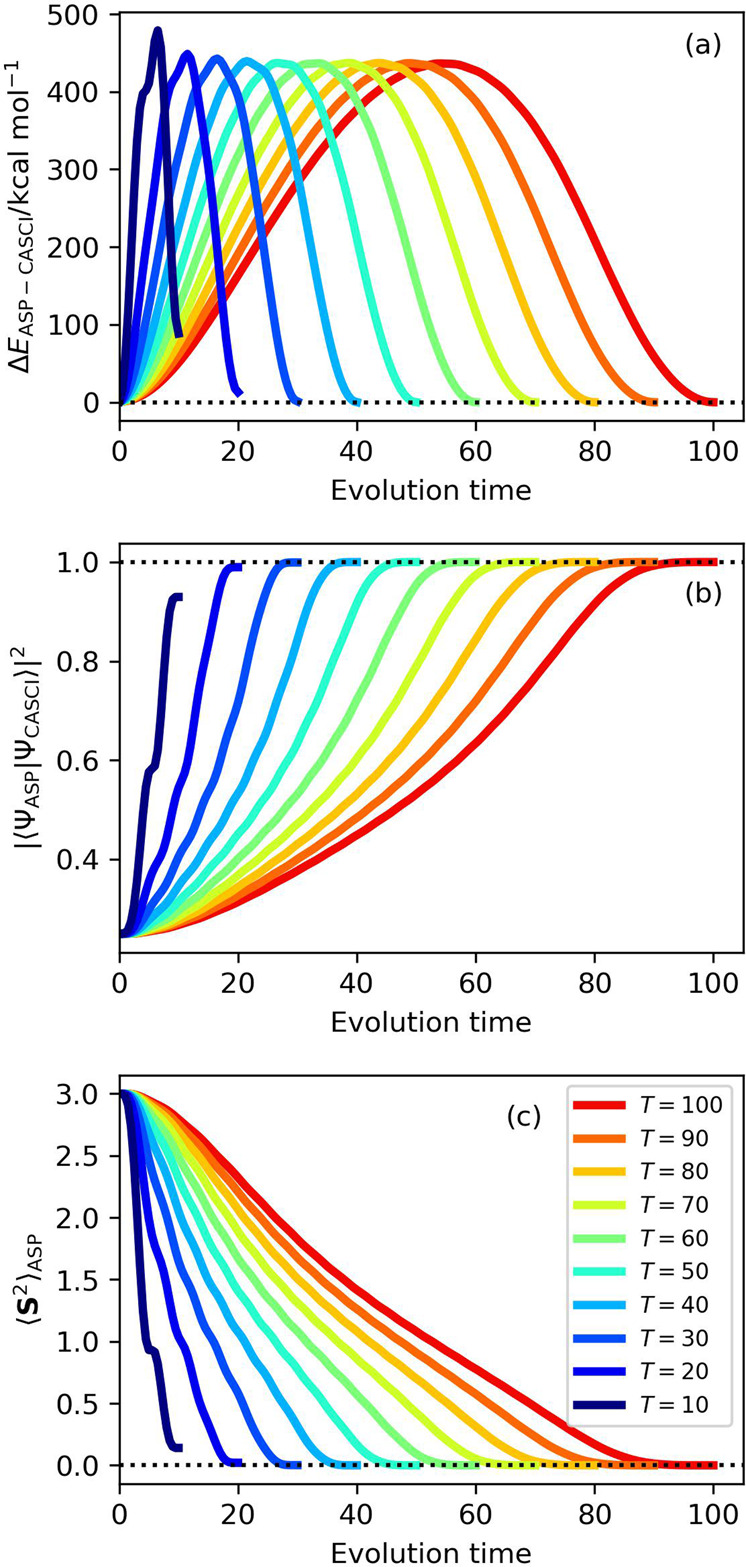
Results of the numerical simulation of ASP with |Ψ_BS_〉 as the starting wave function and square function as the scheduling function in N_2_ molecule at R(N–N) = 3.0 Å. **a** The energy deviations from the CASCI values. **b** The square overlaps with the CASCI wave functions. **c** The 〈**S**^2^〉 values.

**Fig. 7:**
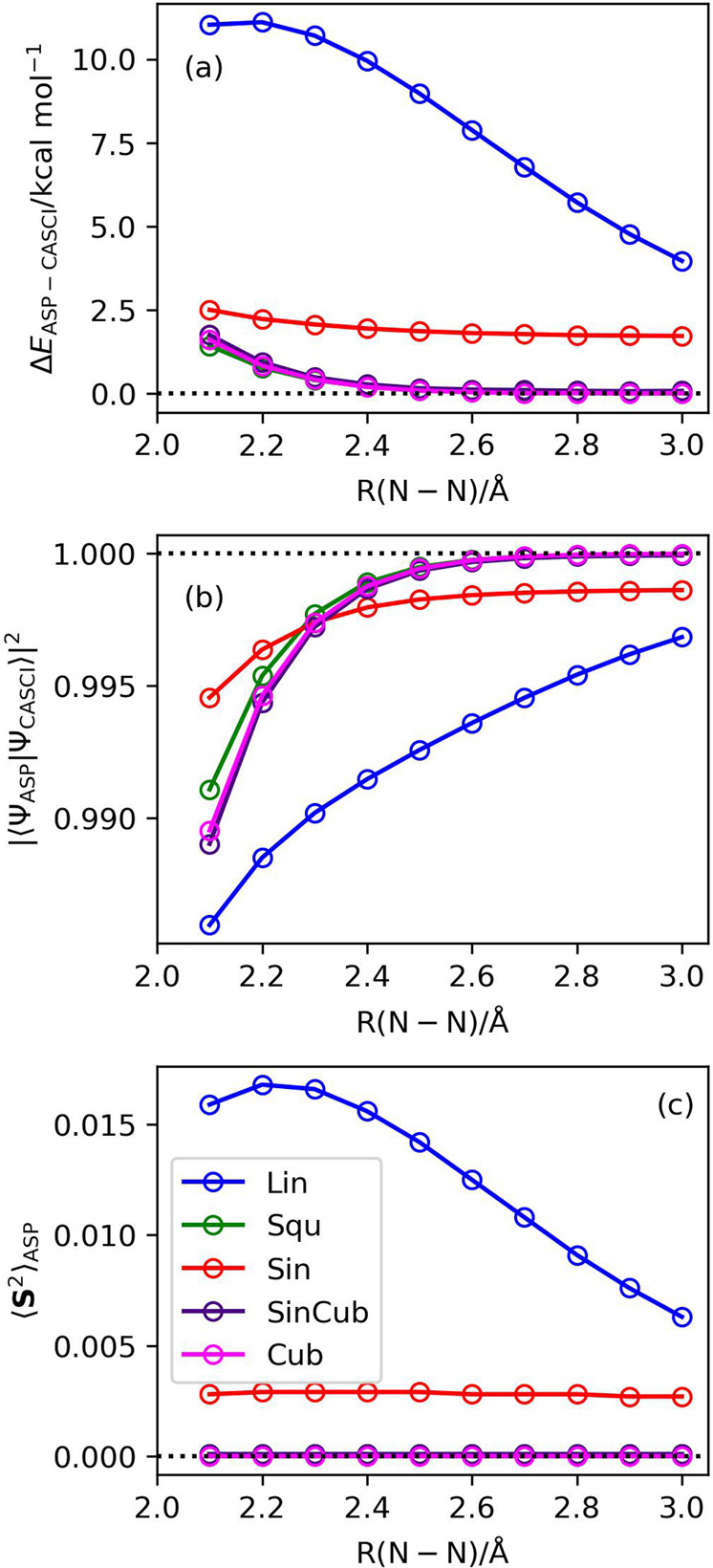
Numerical simulation results of the scheduling function dependence on ASP with the |Ψ_BS_〉 as the starting wave function and *T* = 50 in N_2_ molecule. **a** The energy deviations from the CASCI values. **b** The square overlaps with the CASCI wave functions. **c** The 〈**S**^2^〉 values.

The results of the ASP starting from the six-spin |Ψ_BS_〉 (the wave function in Eq. 4) with *c* = 0.5, the square scheduling function, and the evolution time length *T* = 10–100 of N_2_ molecule at R(N–N) = 3.0 Å are summarized in Fig. 6, and the results obtained by using the other scheduling functions are given in Supplementary Fig. S3–S6. Interestingly, ASP starting with the |Ψ_BS_〉 gave the square overlap |〈Ψ_ASP_|Ψ_CASCI_〉|^2^ larger than 0.995 even for *T* = 20. The scheduling function dependence on ASP with the |Ψ_BS_〉 with the total evolution time *T* = 50 were plotted in Fig. 7. The |〈Ψ_ASP_|Ψ_CASCI_〉|^2^ values calculated by using the square, sinusoidal cubic, and cubic scheduling functions asymptotically approach unity for longer N–N distances. These three scheduling functions exhibit the best square overlap values in the bond dissociation region. No significant differences were observed among these scheduling functions, but the square function gave larger square overlap than the sinusoidal cubic and cubic functions for shorter N–N bond lengths. Importantly, the square, sinusoidal cubic, and cubic scheduling functions gave almost spin-pure wave functions with 〈**S**^2^〉_ASP_ ≈ 0, although spin contaminations were not eliminated completely in the |Ψ_ASP_〉 generated by employing the sinusoidal and linear scheduling functions. In the following discussions we used the square scheduling function for ASP with the |Ψ_BS_〉 wave functions.

Again, the reason why the square, sinusoidal cubic, and cubic scheduling functions gave good results can be explained by the structure of wave functions and the energy landscape of the adiabatic evolution pathway. As given in Eqs. 3 and 4, the |Ψ_BS_〉 is expressed by the linear combination of electronic states belonging to different spin multiplicities. These electronic states have similar energies in the beginning of ASP, but the quasi-degeneracy is gradually lifted as the ASP proceeds, owing to the **S**^2^ operator introduced as the penalty term in the Hamiltonian. Therefore, variation of the Hamiltonian must slow in the earlier stage of ASP, if the |Ψ_BS_〉 is used as the starting Hamiltonian. All the square, sinusoidal cubic, and cubic functions have zero gradient d*s*(*x*)/d*x* = 0 at *x* = 0, which is very important to acquire good wave functions in the ASP with the |Ψ_BS_〉.

Note that ASP with the |Ψ_BS_〉 has not only advantages described above but also disadvantages. The BS method generally breaks the spatial symmetry as well as the spin symmetry, because the localized orbitals are prepared by taking a linear combination of HOMO – *i* and LUMO + *i* orbitals. In the ASP starting from |Ψ_HF_〉, we can utilize the point group symmetry to reduce the number of the nonzero Hamiltonian terms, but such reduction is not applicable for the |Ψ_BS_〉 with localized MOs. In addition, the computational cost of the time evolution operator under the **S**^2^ operator, exp(−*i***S**^2^*t*) also pushes the computational cost of ASP. The scaling of the computational cost of simulating exp(−*i***S**^2^*t*) is *O*(*n*_MO_^2^*M*), where *n*_MO_ is the number of molecular orbitals in the active space and *M* refers to the number of ASP steps. As a result, the computational cost of each ASP step is larger by using the |Ψ_BS_〉 than that for using the |Ψ_HF_〉, especially for the molecular systems with high point group symmetry. However, for large molecular systems, it is possible to take advantage of the locality of MOs to reduce the computational cost, by ignoring the Hamiltonian terms having the norms smaller than the threshold^44^. It should be also noted that our numerical simulations suggest QITE can be also accelerated by using the |Ψ_BS_〉 as the starting wave function in conjunction with the **S**^2^ penalty term in the Hamiltonian.

### A criterion for the switching of the starting wave function

In the previous section, we demonstrated that ASP using the |Ψ_BS_〉 converges quickly in the region of the bond dissociation. However, we can expect that the |Ψ_HF_〉 is more suitable for the starting wave function of ASP around the equilibrium geometry. It should be also noted that the BS-UHF calculation converges to the RHF solution for shorter bond lengths. Constructing a guideline for the switching of the starting wave function is important toward practical application of ASP to other molecular systems.

Here, we have examined a criterion for selecting the starting wave function based on diradical characters *y*^45,46^. Diradical characters are used as the measure of open shell electronic configurations. At the spin-projected UHF level, they can be calculated from the occupation number of the unoccupied natural orbitals *n*_LUNO+*i*_, using Eq. 6^46^.

6 $${y}_{i}=1-\frac{2\left(1-{n}_{{{{{{\rm{LUNO}}}}}}+i}\right)}{1+{\left(1-{n}_{{{{{{\rm{LUNO}}}}}}+i}\right)}^{2}}$$

In the triple bond dissociation of N_2_ molecule, two types of diradical characters can have significant values: *y*(π) = *y*_0_ = *y*_1_ and *y*(σ) = *y*_2_, those reflect open shell electronic configurations in the valence π and σ bonds, respectively. Thus, there are two possible choices of the |Ψ_BS_〉 in the ASP study of the potential energy curve of N_2_ molecule, |Ψ_BS2_〉 and |Ψ_BS3_〉. The |Ψ_BS2_〉 is the four-spin BS wave function where two π orbitals are treated by means of the BS approach. The |Ψ_BS3_〉 is a six-spin BS wave function and both the σ and π orbitals are dealt with the BS framework.

**Fig. 8:**
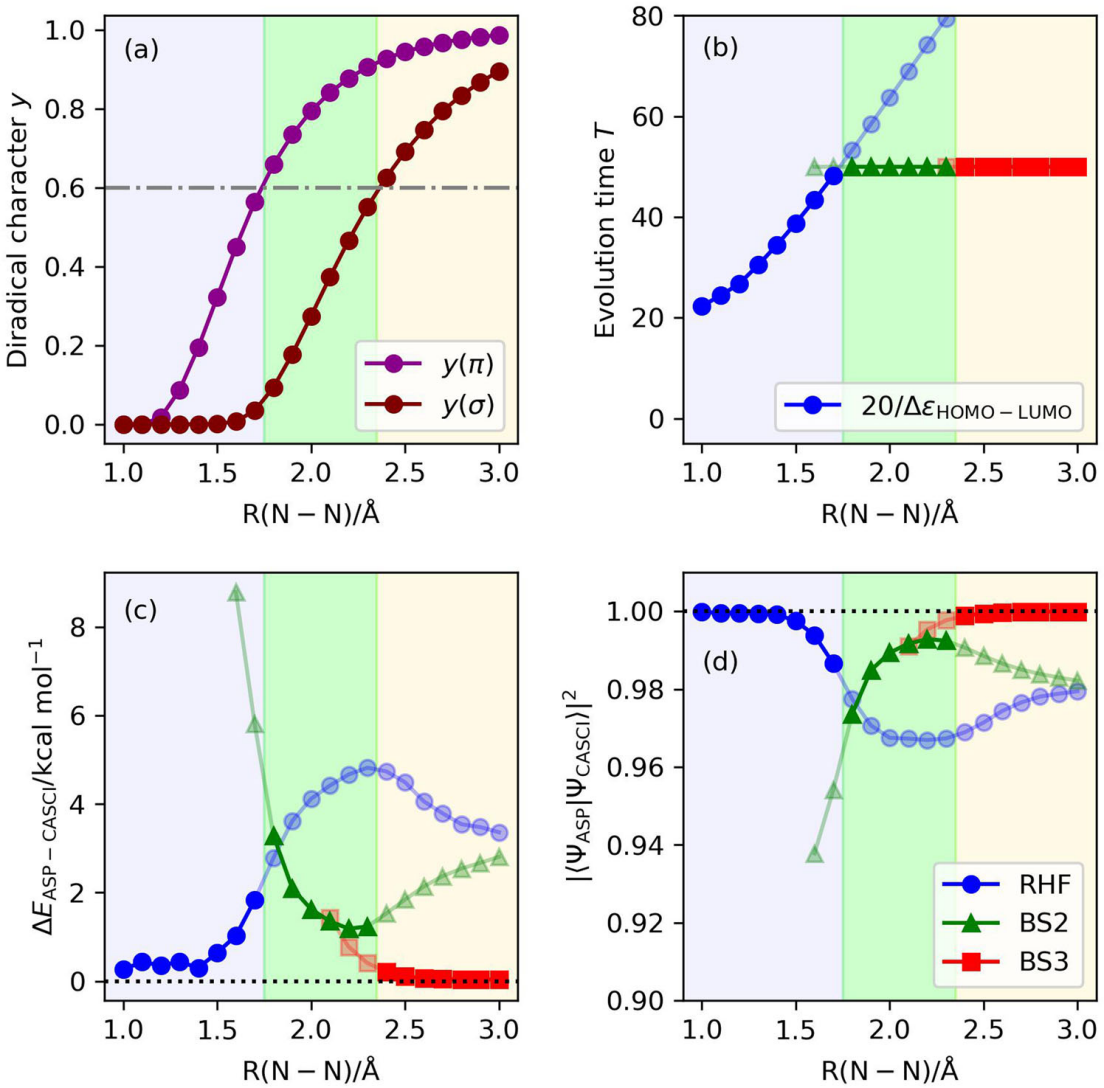
Results of the numerical simulation of ASP of the potential energy curve for N_2_ molecule, using the diradical characters as the indicator for selecting the initial wave function. Background colors specify the regions of the initial wave function recommended from the criterion based on the diradical character *y* > 0.6. Sinusoidal and square functions were adopted as the scheduling functions with the |Ψ_HF_〉 and |Ψ_BS_〉, respectively, as the starting wave function. **a** Diradical characters calculated using Eq. 6. **b** Evolution time lengths. **c** The energy differences from the CASCI values. (d) The square overlaps with the CASCI wave functions.

We assume that the BS treatment is more feasible if the corresponding diradical character is larger. Following this assumption, we explored the threshold value of the diradical characters for the switching of the starting wave function. By setting the evolution time length *T* = 20/Δ*ε* for the ASP with the |Ψ_HF_〉 and *T* = 50 for that with the |Ψ_BS_〉, we found that setting the threshold value for the diradical characters to be 0.6 gives fairly good results, from the viewpoints of both the evolution time length and quality of the |Ψ_ASP_〉, in the potential energy curve of N_2_ molecule (Fig. 8). This is the result of particular ASP conditions, N_2_ molecule with (6e,6o) active space and STO-3G basis set with the sinusoidal and square scheduling functions for the |Ψ_HF_〉 and the |Ψ_BS_〉, respectively, and it is natural that different molecular systems and different computational conditions will give different optimal threshold values for the switching of the starting wave function. We also carried out the ASP simulations of N_2_ molecule using the 6-31G* and 6-311G* basis sets with (6e,6o), (10e,8o), (6e,8o), and (10e,10o) active spaces, obtaining qualitatively the same results (see Supplementary Note 4). Nevertheless, the exploration of other molecular systems is necessary to get further insight of ASP. In the following sections, we describe the results of ASP in the potential energy curve under the symmetric Be–H bond dissociation in the linear BeH_2_ molecule, and the *C*_2*v*_ quasi-reaction pathway of the Be + H_2_ → BeH_2_ reaction.

### Potential energy curve of the symmetric bond dissociation in the linear BeH_2_

**Fig. 9:**
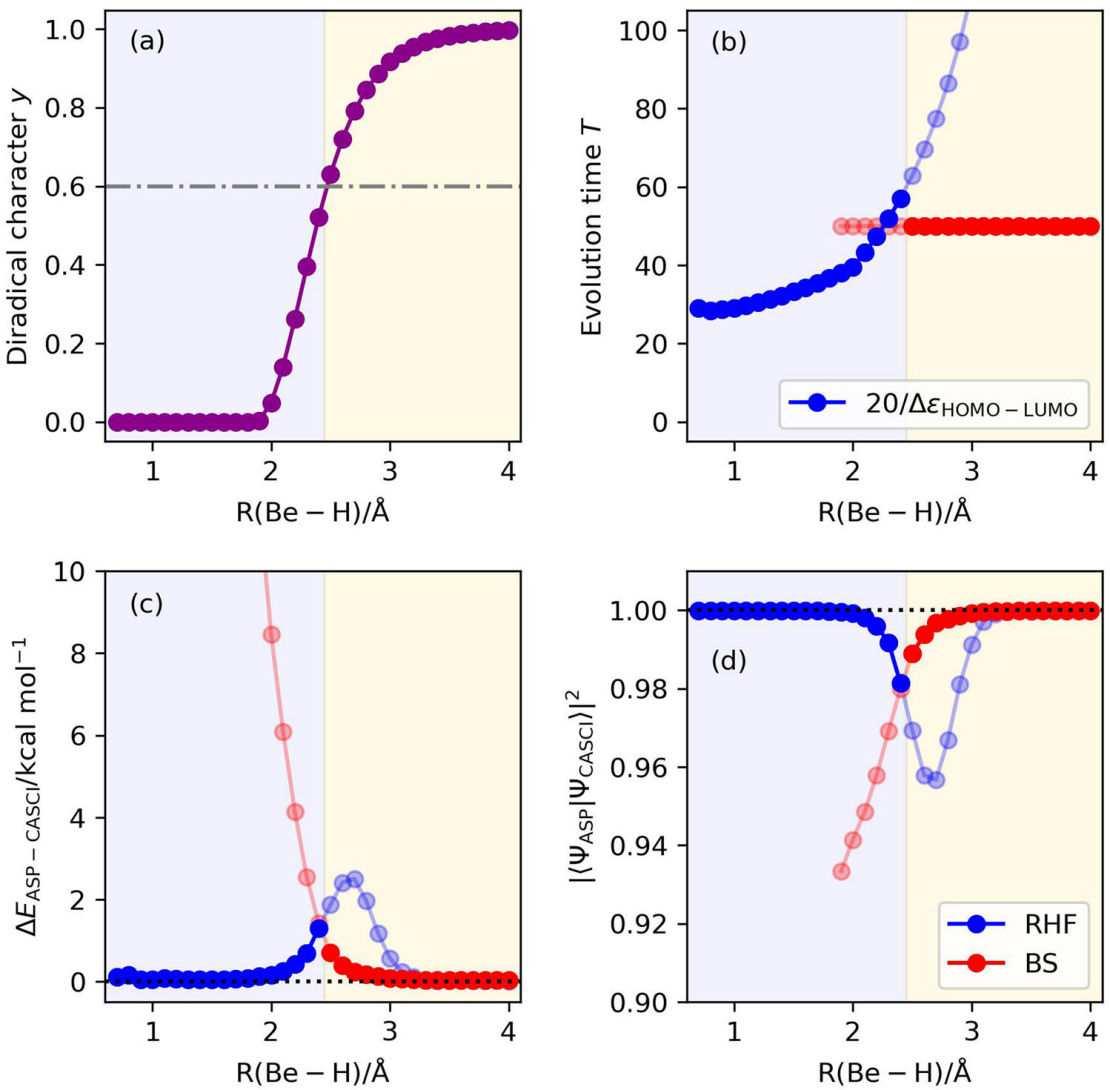
Results of the numerical simulation of ASP of the potential energy curve of the symmetric bond dissociation in BeH_2_ molecule, using the diradical character as the indicator for selecting the initial wave function selection. Background colors specify the regions of the initial wave function recommended from the criterion based on the diradical character *y* > 0.6. Sinusoidal and square functions were adopted as the scheduling functions with the |Ψ_HF_〉 and |Ψ_BS_〉, respectively, as the starting wave function. **a** Diradical characters calculated using Eq. 6. **b** Evolution time lengths. **c** The energy differences from the CASCI values. **d** The square overlaps with the CASCI wave functions.

BeH_2_ is a linear molecule with R(Be–H) = 1.326 Å in the equilibrium geometry^47^. Symmetric elongation of two Be–H bonds generates a Be atom in the (1s)^2^(2s)^2^ closed-shell singlet electron configuration and two H atoms. In this work, we have explored the potential energy curve in the range of R(Be–H) from 0.7 to 4.0 Å. The BS-UHF/STO-3G calculations converged to the RHF solution at the bond length R(Be–H) = 1.9 Å and shorter. The calculated diradical character *y* is plotted in Fig. 9a. By adopting the criterion for selecting the starting wave function discussed in the previous section, we expect that the |Ψ_BS_〉 is suitable for the starting wave function of ASP at the bond length R(Be–H) = 2.5 Å and longer. The quantum circuit simulation results are summarized in Fig. 9. By switching the starting wave function from the |Ψ_HF_〉 to the |Ψ_BS_〉 at the bond length R(Be–H) = 2.5 Å, we obtained the |Ψ_ASP_〉 with the square overlap |〈Ψ_ASP_|Ψ_CASCI_〉|^2^ > 0.98 for all the bond lengths being studied. These results exemplify the usefulness of the diradical character as the indicator of the starting wave function switching. However, the |〈Ψ_ASP_|Ψ_CASCI_〉|^2^ values are slightly larger in the geometries with intermediate diradical characters (R(Be–H) ~ 2.4 Å). The ASP simulations with the longer evolution time revealed that we can achieve |〈Ψ_ASP_|Ψ_CASCI_〉|^2^ > 0.998 for *T* = 200 and 100 with |Ψ_HF_〉 and |Ψ_BS_〉, respectively, as the starting wave function (see Supplementary Figs. S16 and S17). We also examined the numerical simulations by using the other scheduling functions for ASP with the |Ψ_HF_〉 and the evolution time length *T* = 20/Δ*ε*. The results are summarized in Supplementary Fig. S18, insisting that the sinusoidal function is suitable for the scheduling function.

### The *C*_2*v*_ quasi-reaction pathway of Be atom insertion to H_2_

Finally, we have examined ASP in the *C*_2*v*_ quasi-reaction pathway of the Be atom insertion to H_2_ molecule. This system has been widely studied as the model of strongly correlated electronic structures^48–52^. Cartesian coordinates of H atoms are summarized in Table 2. The reaction pathway contains the S_0_–S_1_ avoided crossing at the transition structure (point E in Table 2), and the energy gap between the S_0_ and S_1_ states becomes small around the transition structure.

**Table 2:** Cartesian coordinates of H atoms for the points being studied in the Be + H_2_ → BeH_2_ reaction, in units of Bohr^[*a*]^.

Point	X	Y	Z
A	0.000	±2.540	0.000
B	0.000	±2.080	1.000
C	0.000	±1.620	2.000
D	0.000	±1.390	2.500
E	0.000	±1.275	2.750
F	0.000	±1.160	3.000
G	0.000	±0.930	3.500
H	0.000	±0.700	4.000
I	0.000	±0.700	6.000

^[*a*]^Be atom is located at the origin of coordinates.

**Fig. 10:**
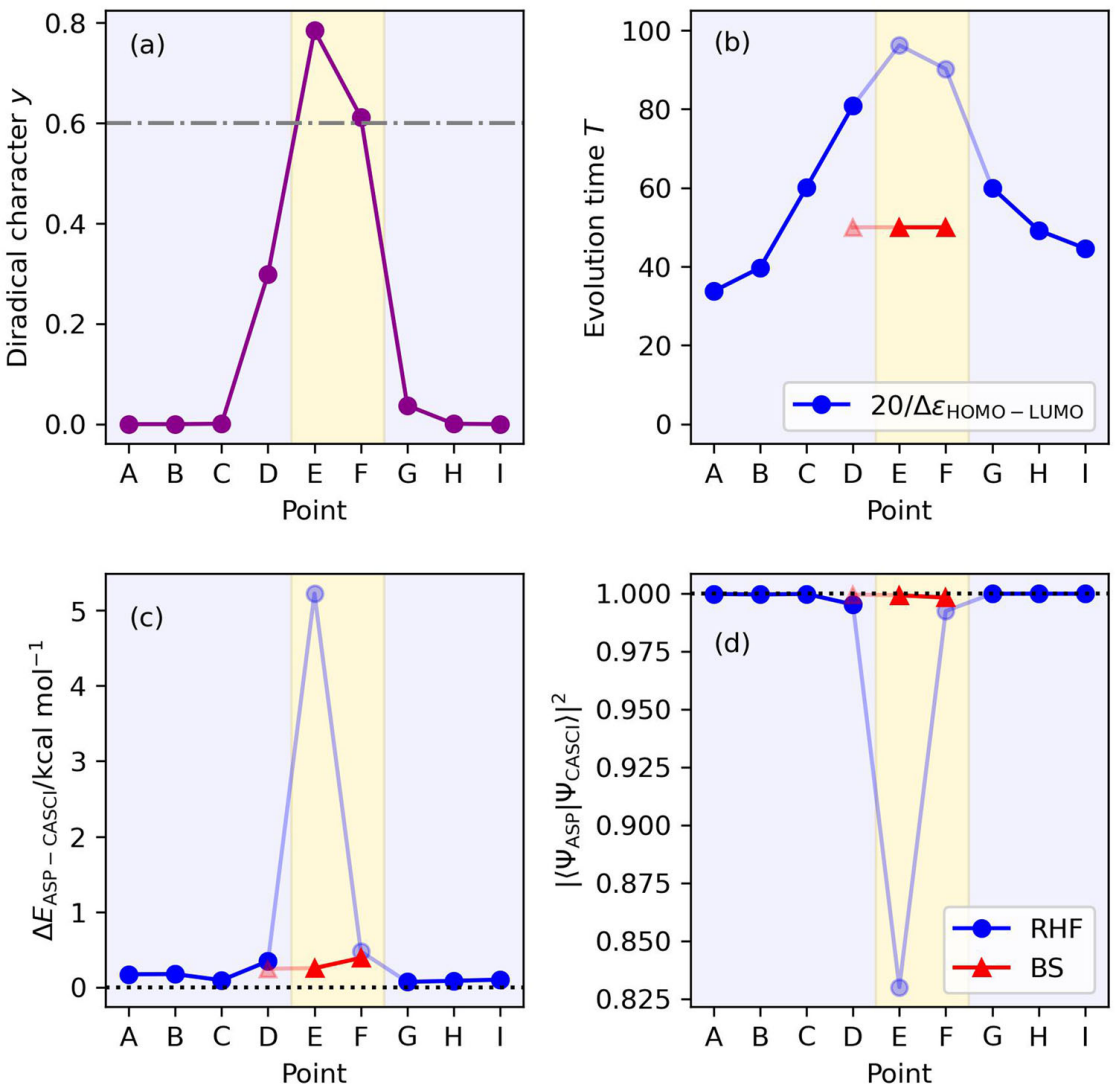
Results of the numerical simulation of ASP of the *C*_2*v*_ quasi-reaction pathway of Be + H_2_ → BeH_2_, using the diradical character as the indicator for selecting the initial wave function. Background colors specify the regions of the initial wave function recommended from the criterion based on the diradical character *y* > 0.6. Sinusoidal and square functions were adopted as the scheduling functions with the |Ψ_HF_〉 and |Ψ_BS_〉, respectively, as the starting wave function. **a** Diradical characters calculated using Eq. 6. **b** Evolution time lengths. **c** The energy differences from the CASCI values. **d** The square overlaps with the CASCI wave functions.

Results of the numerical quantum circuit simulations are summarized in Fig. 10. By using the |Ψ_HF_〉 as the starting wave function and setting the evolution time length as *T* = 20/Δ*ε* and using the sinusoidal scheduling function, we obtained the correlated wave function with the square overlap close to unity, except for point E. To disclose the convergence behavior of ASP at point E, we examined the ASP simulations with longer evolution time lengths *T* = 200, 300, 400, and 500. The square overlaps were calculated to be |〈Ψ_ASP_|Ψ_CASCI_〉|^2^ = 0.9308, 0.9720, 0.9894, and 0.9962, respectively, and thus the convergence of the square overlap against the evolution time length *T* is very slow. The simulations around point E with finer geometrical changes revealed that the square overlap is smaller for the geometry closer to the transition structure (see Supplementary Note 7). We also computed the energy landscape of the instantaneous Hamiltonian at point E, finding that the S_1_ and S_0_ states become almost gapless around *s*(*x*) = 0.9 (see Supplementary Fig. S19). The 16, 14, and 12 qubit ASP simulations by removing the highest virtual orbitals one by one gave the square overlaps |〈Ψ_ASP_|Ψ_CASCI_〉|^2^ = 0.8464, 0.8538, and 0.9751, respectively. These results exemplify that selecting appropriate active orbitals is essential to obtain sophisticated wave functions from ASP. In fact, 16 qubit ASP simulations using the natural orbitals constructed from CISD/6-31G* calculations gave the square overlap larger than 0.98 for all points being studied (see Supplementary Fig. S21).

The BS-UHF calculations revealed that points D, E, and F have non-negligible diradical characters. The diradical characters were calculated to be *y* = 0.2991, 0.7851, and 0.6125 for points D, E, and F, respectively. The diradical character indicates that the |Ψ_BS_〉 is suitable for the starting wave function of ASP in points E and F. Our numerical simulations revealed that ASP with the |Ψ_BS_〉 gave larger square overlap values with the CASCI wave functions not only at points E and F but also at point D, with shorter evolution time length than ASP with the |Ψ_HF_〉. More extended studies are necessary to thoroughly understand the performance of ASP, which is left as future work.

## Conclusions

In this work, we have examined the numerical quantum circuit simulations of ASP in N_2_ and BeH_2_ molecules, seeking practical computational conditions for the generation of correlated wave functions having the square overlap with the CASCI wave function close to unity. The numerical quantum circuit simulations revealed that if the |Ψ_HF_〉 is employed as the starting wave function, the sinusoidal function *s*(*x*) = sin(π*x*/2), where *x* = *t*/*T*, gives the wave function having large square overlaps for shorter evolution time lengths among the five scheduling functions being tested. By using the |Ψ_HF_〉 as the starting wave function, the evolution time length required to achieve sufficiently large square overlap with the CASCI wave function increases with increasing the open-shell character and decreasing the HOMO−LUMO gap. By setting the evolution time length *T* = 20/Δ*ε*, where Δ*ε* denotes the HOMO−LUMO gap, we can obtain the correlated wave function with the square overlap larger than 0.95, except for point E as given in the Be + H_2_ → BeH_2_ reaction pathway. By contrast, by using the |Ψ_BS_〉 as the starting wave function and introducing the **S**^2^ operator as the penalty term in the time-dependent Hamiltonian, the ASP simulations with the square function *s*(*x*) = 3*x*^2^ − 2*x*^3^, sinusoidal cubic function *s*(*x*) = sin^3^(π*x*/2), and cubic function *s*(*x*) = 6*x*^5^ − 15*x*^4^ + 10*x*^3^ gave the correlated wave function with the square overlap close to unity with substantially shorter evolution time length than ASP with the |Ψ_HF_〉 when the diradical character *y* is large. The results of the present numerical quantum circuit simulations indicate that ASP is capable of generating the correlated wave functions with sufficiently large square overlap with the CASCI wave function by switching the starting wave function from the |Ψ_HF_〉 to the |Ψ_BS_〉 and simultaneously changing the scheduling function from the sinusoidal to the square functions, when the diradical character exceeds 0.6. ASP numerical simulations with larger basis sets revealed the importance of the appropriate active space selections based on the occupation number of natural orbitals to acquire sophisticated wave functions from ASP.

It should be emphasized that the computational conditions described in this paper do not have to be optimal for any molecular systems. The number of theoretical and experimental studies of ASP is not many and more elaborated investigations are necessary to shed light on the performance of ASP. Another important direction of the study of ASP is to connect to the QPE algorithms including the Bayesian phase difference estimation algorithm, which is a controlled-time evolution-free algorithm^5,53^. The relevant studies are underway and will be published in the forthcoming paper.

## Methods

### Adiabatic state preparation

Assume that the quantum system is in the ground state of an initial Hamiltonian *H*_*I*_ at *t* = 0, and the Hamiltonian of the system changes slowly. If the variation of the Hamiltonian is sufficiently slow, the system remains in the ground state of the instantaneous Hamiltonian at *t* > 0, which is known as an adiabatic theorem^19^. ASP utilizes the adiabatic theorem to obtain the full-CI wave function, by using the Fock operator *F* defined in Eq. 7 as *H*_*I*_ and the electronic Hamiltonian *H* in Eq. 8 as *H*_*P*_.

7 $$F=\mathop{\sum}\limits_{i}{h}_{{ii}}{a}_{i}^{{{\dagger}} }{a}_{i}+\frac{1}{2}\mathop{\sum}\limits_{{ij}}\left({h}_{{ijji}}{a}_{i}^{{{\dagger}} }{a}_{j}^{{{\dagger}} }{a}_{j}{a}_{i}+{h}_{{ijij}}{a}_{i}^{{{\dagger}} }{a}_{j}^{{{\dagger}} }{a}_{i}{a}_{j}\right)$$

8 $$H=\mathop{\sum}\limits_{{pq}}{h}_{{pq}}{a}_{p}^{{{\dagger}} }{a}_{q}+\frac{1}{2}\mathop{\sum}\limits_{{pqrs}}{h}_{{pqrs}}{a}_{p}^{{{\dagger}} }{a}_{q}^{{{\dagger}} }{a}_{r}{a}_{s}$$

Throughout this paper, we used indices *i* and *j* for the occupied spin orbitals in the starting wave function, *a* and *b* for the unoccupied spin orbitals, and *p*, *q*, *r*, and *s* for general spin orbitals. *h*_*pq*_ and *h*_*pqrs*_ are one- and two-electron molecular orbital (MO) integrals defined in Eqs. 9 and 10, respectively. *a*_*p*_^†^ and *a*_*p*_ are creation and annihilation operators, respectively, acting on the *p*th spin orbital.

9 $${h}_{{pq}}=\int {\phi }_{p}^{* }\left({{{{{\boldsymbol{r}}}}}}\right)\left(-\frac{1}{2}{\nabla }^{2}-\mathop{\sum}\limits_{A}\frac{{Z}_{A}}{\left|{{{{{\boldsymbol{r}}}}}}-{{{{{{\boldsymbol{R}}}}}}}_{A}\right|}\right){\phi }_{q}\left({{{{{\boldsymbol{r}}}}}}\right)d{{{{{\boldsymbol{r}}}}}}$$

10 $${h}_{{pqrs}}=\iint {\phi }_{p}^{* }\left({{{{{\boldsymbol{r}}}}}}\right){\phi }_{q}^{* }\left({{{{{\boldsymbol{r}}}}}}^{{{\prime} }}\right)\frac{1}{\left|{{{{{{\boldsymbol{r}}}}}}}^{{{{\prime} }}}-{{{{{\boldsymbol{r}}}}}}\right|}{\phi }_{r}\left({{{{{\boldsymbol{r}}}}}}^{{{\prime} }}\right){\phi }_{s}\left({{{{{\boldsymbol{r}}}}}}\right)d{{{{{\boldsymbol{r}}}}}}d{{{{{\boldsymbol{r}}}}}}^{{{\prime} }}$$

In Eq. 9, *A* runs over atoms, and *Z*_*A*_ and ***R***_*A*_ are the atomic number and spatial coordinates, respectively, of atom *A*. *ϕ*_*p*_ is the spatial part of the spin orbital of *p*.

In order to implement ASP on a gate model quantum computer, the adiabatic evolution is usually discretized: the evolution time length *T* is divided into *M* steps, and the time evolution under the time-independent Hamiltonian *H*_*m*_ in Eqs. 11 and 12 is simulated.

11 $$|\Psi (T)\rangle ={e}^{-i{H}_{M}t/M}\cdots {e}^{-i{H}_{2}t/M}\cdot {e}^{-i{H}_{1}t/M}|{\Psi }_{I}\rangle$$

12 $${H}_{m}=\left(1-s\left(m/M\right)\right){H}_{I}+s\left(m/M\right){H}_{P}$$

There are several error sources in ASP. For example, if the evolution time length *T* is too short to follow the adiabatic path or if the step number *M* is too small and the Hamiltonian of the (*m* + 1)th step is too different from that of the *m*th step, the probability to cause nonadiabatic transitions to other electronic states becomes large and the wave function obtained from ASP is expected to have smaller overlap with the exact wave function. Error sources inherent in quantum computing such as decoherence and errors arising from Trotter decomposition, as discussed in the next section, also affect the quality of the wave function. Note that the computational cost of ASP increases linearly to the step number *M*, although the finer time steps generally give the wave function with the larger overlap with the exact wave function.

### Quantum chemical calculations on a quantum computer

To execute ASP on a gate model quantum computer, wave functions are mapped onto qubits by using a fermion–qubit transformation technique, and the quantum circuit corresponding to the time evolution operator is constructed by using basic quantum gate sets^54^. Several fermion–qubit transformation techniques have been proposed^55–58^, and in this work we adopted a Jordan–Wigner transformation (JWT)^4,55^. In the JWT, the wave function of the *N* spin-orbital systems is mapped onto *N* qubits, and each qubit stores an occupation number of a particular spin orbital: the qubit is in the |1〉 state if the corresponding spin orbital is occupied by an electron, otherwise in the |0〉 state. The creation and annihilation operators appearing in the second quantized Hamiltonian (Eqs. 7 and 8) are transformed onto the direct products of Pauli operators (Pauli strings) using Eqs. 13 and 14, respectively.

13 $${a}_{p}^{{{\dagger}} }=\frac{1}{2}\left({X}_{p}-i{Y}_{p}\right)\otimes {\prod }_{r=1}^{p-1}{Z}_{r}$$

14 $${a}_{p}=\frac{1}{2}\left({X}_{p}+i{Y}_{p}\right)\otimes {\prod }_{r=1}^{p-1}{Z}_{r}$$

Here, *X*_*p*_, *Y*_*p*_, and *Z*_*p*_ are Pauli operators defined in Eqs. 15–17, acting on the *p*th qubit.

15 $$X=\left(\begin{array}{cc}0 & 1\\ 1 & 0\end{array}\right)$$

16 $$Y=\left(\begin{array}{cc}0 & -i\\ i & 0\end{array}\right)$$

17 $$Z=\left(\begin{array}{cc}1 & 0\\ 0 & -1\end{array}\right)$$

By applying the JWT, the second quantized Hamiltonians in Eqs. 7 and 8 are transformed onto qubit Hamiltonians consisting of a linear combination of Pauli strings, as in Eqs. 18 and 19.

18 $$H=\mathop{\sum }\limits_{k=1}^{K}{\omega }_{k}{P}_{k}$$

19 $${P}_{k}={\sigma }_{N}\otimes {\sigma }_{N-1}\otimes \cdots \otimes {\sigma }_{1},\sigma \in \left\{I,\,X,\,Y,\,Z\right\}$$

The time evolution operator *U* is defined as in Eq. 20. Trotter–Suzuki decomposition^59,60^ is usually used to decompose the time evolution operator and to construct the corresponding quantum circuits. The time evolution operators obtained by applying the first- and second-order Trotter–Suzuki decompositions are given in Eqs. 21 and 22, respectively^61^.

20 $$U={{\exp }}\left(-{iHt}\right)={{\exp }}\left(-i{\sum }_{k=1}^{K}{\omega }_{k}{P}_{k}t\right)$$

21 $$U\approx {\left[{\prod }_{k=1}^{K}{{\exp }}\left(-i{\omega }_{k}{P}_{k}t/L\right)\right]}^{L}$$

22 $$U\approx {\left[{\prod }_{k=1}^{K}{{\exp }}\left(-i{\omega }_{k}{P}_{k}t/2L\right){\prod }_{k=K}^{1}{{\exp }}\left(-i{\omega }_{k}{P}_{k}t/2L\right)\right]}^{L}$$

Note that magnitude of the Trotter decomposition error depends on the ordering of terms. It is known that the magnitude ordering in which Hamiltonian terms are applied in the descending order of the absolute value of the coefficient |*ω*_*k*_| often gives smaller Trotter decomposition errors than the lexicographical ordering that is an ordering scheme, which maximizes the similarity of the Pauli strings of adjacent terms^62,63^. Once the Trotter decomposition is applied, the quantum circuit corresponding to the operator exp(−*iω*_*k*_*P*_*k*_*t*/*L*) is constructed by following the literature^54^. Supplementary Fig. S1 illustrates the quantum circuit corresponding to the operator exp(−*iω*X_0_Z_1_Z_2_X_3_*t*) as an example. Definitions of the quantum gates are also given in the Supplementary Note 1.

### Implementation of ASP

ASP can be implemented by the following procedures. (1) Perform the RHF or BS-UHF calculations using conventional quantum chemistry program packages and compute the molecular integrals defined in Eqs. 9 and 10. (2) Divide the electronic Hamiltonian (Eq. 8) into the Fock operator (Eq. 7) and the rest terms, and apply fermion–qubit transformation to them to obtain corresponding qubit Hamiltonians. (3) Set the computational conditions for ASP. Select the initial wave function, scheduling function for adiabatic evolution, total evolution time, time for the single Trotter step, strategy for Trotter term ordering, and so on. (4) Compute the instantaneous Hamiltonian at each discretized time step, and construct the quantum circuit corresponding to the time evolution operator. (5) Encode the starting wave function on the *N*_orb_ of qubits, where *N*_orb_ is the number of spin orbitals in the active space, and execute the quantum circuit constructed in the step 4.

The steps 1–4 above are performed on a classical computer, and the step 5 is the main part of the ASP computation. If we adopted |Ψ_HF_〉 as the starting wave function and the JWT for fermion–qubit transformation, the wave function encoding described in the step 5 can be done by applying the Pauli-*X* (NOT) gates to the qubits storing the occupation number of occupied orbitals in the |Ψ_HF_〉 to the quantum states initialized to the |00…0〉 state.

Note that in most of adiabatic quantum computing problems the initial Hamiltonian contains the terms those are not included in the final Hamiltonian. On the contrary, all the terms in the Fock operator in Eq. 7 are included in the electronic Hamiltonian in Eq. 8. Therefore, the instantaneous Hamiltonian *H*(*m*) defined in Eq. 12 can be rewritten as in Eq. 23, where Fock specifies the qubit Hamiltonian corresponding to the Fock operator.

23 $$H\left(m\right)=\mathop{\sum}\limits_{{P}_{k}\in {{{{{\rm{Fock}}}}}}}{\omega }_{k}{P}_{k}+\mathop{\sum}\limits_{{P}_{l}\notin {{{{{\rm{Fock}}}}}}}s\left(m/M\right){\omega }_{l}{P}_{l}$$

In the quantum circuit level, the quantum circuit at each time step has exactly the same structure with different rotational angles *θ* of the *R*_*z*_ gate (see Supplementary Fig. S1).

### Computational conditions

In this work, we have focused on three molecular systems: the potential energy curve of the triple bond dissociation of N_2_ molecule, the symmetric Be–H bond dissociations in linear BeH_2_ molecule, and the *C*_2*v*_ quasi-reaction pathway of a Be atom insertion to a H_2_ molecule, at the CASCI level of theory. For the study of the potential energy curve of N_2_ molecule under the triple bond dissociation, we used the STO-3G basis set in conjunction with the six electrons in the six orbital (6e, 6o) active space consisting of valence σ/σ* and π/π* orbitals. The potential energy curve of BeH_2_ molecule under the symmetric Be–H bond cleavage was studied by using the STO-3G basis set and the full-valence (4e, 6o) active space. The quasi-reaction pathway of Be + H_2_ → BeH_2_ was investigated by using the basis set comprised of (10s 3p)/[3s 1p] for Be and (4s)/[2s] for H, which was used by Purvis and coworkers^44^ for the study of the same system. Frozen core orbital approximation was adopted for the CASCI calculations and thus the active space is (4e, 9o).

For the numerical quantum circuit simulations of ASP executable on classical computers, we developed a python program by utilizing Cirq^64^ and OpenFermion^65^ libraries. The step number *M* in Eqs. 11 and 12 was set to be $M=\lceil 2T\rceil$ using a ceil function, and the quantum circuit for the time evolution operator of each step was constructed by adopting the second-order Trotter–Suzuki decomposition with *L* = 1 in Eq. 22. We used the magnitude ordering for the Trotterized time evolution operators, and the ordering of the terms were optimized for every time step.

For the preparation of the starting wave functions of ASP, we performed the RHF and the BS-UHF calculations using GAMESS-US program package^66^. One- and two-electron atomic orbital integrals were also computed using GAMESS-US software, and MO integrals *h*_*pq*_ and *h*_*pqrs*_ in Eqs. 9 and 10 were generated by using our own AO → MO integral transformation program.

## Supplementary information

Supplementary Information

## Data Availability

The data that support the findings on this study are available from the corresponding authors on reasonable request.

## Code availability

The python source code for the numerical quantum circuit simulations of ASP and integral files are available via github repository: https://github.com/Kenji-Sugisaki/ASP.
