# Supplementary material for: Adiabatic state preparation of correlated wave functions with nonlinear scheduling functions and broken-symmetry wave functions
Source: Commun Chem. 2022 Jul 25;5:84. doi: 10.1038/s42004-022-00701-8 (PMC9814591; doi:10.1038/s42004-022-00701-8)
Supplement: Supplementary file 1 — Supplementary Information [file 42004_2022_701_MOESM1_ESM.pdf]

Supplementary Information

Adiabatic state preparation of correlated wave functions with nonlinear scheduling functions and broken-symmetry wave functions

Kenji Sugisaki,[1,2,3*] Kazuo Toyota,[1] Kazunobu Sato,[1*] Daisuke Shiomi,[1] and Takeji Takui[1,4*]

[1] *Department of Chemistry and Molecular Materials Science, Graduate School of Science, Osaka City University, 3-3-138 Sugimoto, Sumiyoshi-ku, Osaka 558-8585, Japan*

[2] *JST PRESTO, 4-1-8 Honcho, Kawaguchi, Saitama, 332-0012, Japan*

[3] *Centre for Quantum Engineering, Research and Education (CQuERE), TCG Centres for Research and Education in Science and Technology (TCG CREST), 16th Floor, Omega, BIPL Building, Blocks EP & GP, Sector V, Salt Lake, Kolkata 700091, India*

[4] *Research Support Department/University Research Administrator Centre, University Administration Division, Osaka City University, 3-3-138 Sugimoto, Sumiyoshi-ku, Osaka 558-8585, Japan*

* E-mail: sugisaki@osaka-cu.ac.jp; sato@osaka-cu.ac.jp; takui@sci.osaka-cu.ac.jp

Table of Contents

	Page
**Supplementary Note 1:** Definitions of quantum gates and an example of the quantum circuit corresponding to time evolution operator.	S2
**Supplementary Note 2:** Scheduling function dependence on the ASP wave functions with different evolution time length determination strategies in N_2 molecule.	S4
**Supplementary Note 3:** Scheduling function dependence on the numerical simulation of ASP with the $\vert\Psi_{BS}\rangle$ as the starting wave function in N_2 molecule at R(N–N) = 3.0 Å.	S5
**Supplementary Note 4:** Numerical ASP simulations of N_2 molecule using the 6-31G* and 6-311G* basis sets with (6e,6o), (10e,8o), (6e,8o), and (10e,10o) active spaces.	S7
**Supplementary Note 5:** Results of the numerical simulation of ASP for the BeH_2 potential energy surface with longer evolution times.	S17
**Supplementary Note 6:** Results of the numerical simulation of ASP with different evolution time length determination strategies in BeH_2.	S19
**Supplementary Note 7:** Numerical ASP simulations around the transition states of the Be + H_2 → BeH_2 quasi-reaction pathway.	S20
**Supplementary Note 8:** Numerical ASP simulations of the Be + H_2 → BeH_2 quasi-reaction pathway by using the 6-31G* basis set.	S22

Supplementary Note 1

Definitions of quantum gates and an example of the quantum circuit corresponding to time evolution operator.

In quantum computers, qubits can be in an arbitrary superposition of the $|0\rangle$ and $|1\rangle$ states, as given in Eq. S1.

$$|\varphi\rangle = c_0|0\rangle + c_1|1\rangle \quad \text{(S1)}$$

Here, c_0 and c_1 are arbitrary complex numbers satisfying a normalization condition given in Eq. S2.

$$|c_0|^2 + |c_1|^2 = 1 \quad \text{(S2)}$$

The quantum state $|\varphi\rangle$ in Eq. S1 can also be represented by a matrix as follows:

$$|\varphi\rangle = \begin{pmatrix} c_0 \\ c_1 \end{pmatrix} \quad \text{(S3)}$$

Quantum gates acting on one qubit can be expressed by a (2 × 2) unitary matrix and the quantum state after the quantum gate application can be calculated by matrix algebra. For example, the quantum state after the application of an Hadamard gate can be calculated as in Eq. S4.

$$H_d|\varphi\rangle = \frac{1}{\sqrt{2}}\begin{pmatrix} 1 & 1 \\ 1 & -1 \end{pmatrix}\begin{pmatrix} c_0 \\ c_1 \end{pmatrix} = \frac{1}{\sqrt{2}}\begin{pmatrix} c_0 + c_1 \\ c_0 - c_1 \end{pmatrix} \quad \text{(S4)}$$

The circuit symbols and matrix representations of the quantum gates frequently used for quantum chemical calculations are summarized in Table S1. In the quantum circuit, the horizontal lines denote a qubit or *N*-qubits, and squares, circles, and vertical lines represent quantum gates, which are applied to the qubits from left to right order. Fig. S1 illustrates the quantum circuit corresponding to the operator $\exp(-i\omega X_0Z_1Z_2X_3t)$ as an example. Here, X_p and Z_p are Pauli-X and Z operators acting on the *p*th qubit defined in Eq. 15 and 17, respectively, in the main text.

Table S1. Graph and matrix representations of quantum gates.

Gate	Circuit symbol	Matrix representation
Hadamard (H_d)	H_d	$\frac{1}{\sqrt{2}}\begin{pmatrix}1 & 1\\ 1 & -1\end{pmatrix}$
Pauli-*X*	X	$\begin{pmatrix}0 & 1\\ 1 & 0\end{pmatrix}$
Pauli-*Y*	Y	$\begin{pmatrix}0 & -i\\ i & 0\end{pmatrix}$
Pauli-*Z*	Z	$\begin{pmatrix}1 & 0\\ 0 & -1\end{pmatrix}$
$R_x(\theta)$	$R_x(\theta)$	$\begin{pmatrix}\cos\frac{\theta}{2} & -i\sin\frac{\theta}{2}\\ -i\sin\frac{\theta}{2} & \cos\frac{\theta}{2}\end{pmatrix}$
$R_y(\theta)$	$R_y(\theta)$	$\begin{pmatrix}\cos\frac{\theta}{2} & -\sin\frac{\theta}{2}\\ \sin\frac{\theta}{2} & \cos\frac{\theta}{2}\end{pmatrix}$
$R_z(\theta)$	$R_z(\theta)$	$\begin{pmatrix}e^{-i\theta/2} & 0\\ 0 & e^{i\theta/2}\end{pmatrix}$
Controlled-NOT (CNOT)		$\begin{pmatrix}1 & 0 & 0 & 0\\ 0 & 1 & 0 & 0\\ 0 & 0 & 0 & 1\\ 0 & 0 & 1 & 0\end{pmatrix}$

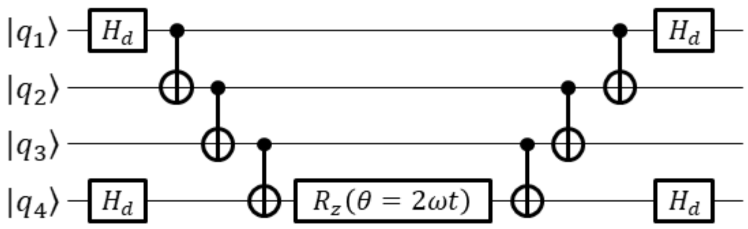

Fig. S1 Quantum circuit corresponding to the time evolution operator $\exp(-i\omega X_0Z_1Z_2X_3t)$.

Supplementary Note 2

Scheduling function dependence on the ASP wave functions with different evolution time length determination strategies in N_2 molecule.

The results of the quantum circuit simulations with three different evolution time length determination strategies (T = $10/\Delta\varepsilon^2$, $5/\Delta\varepsilon^2$, and $20/\Delta\varepsilon$, where $\Delta\varepsilon$ is the HOMO–LUMO energy gap obtained from the RHF/STO-3G wave function) in conjunction with the sinusoidal (Sin), linear (Lin), square (Squ), sinusoidal cubic (SinCub), and cubic (Cub) scheduling functions are summarized in Fig. S2.

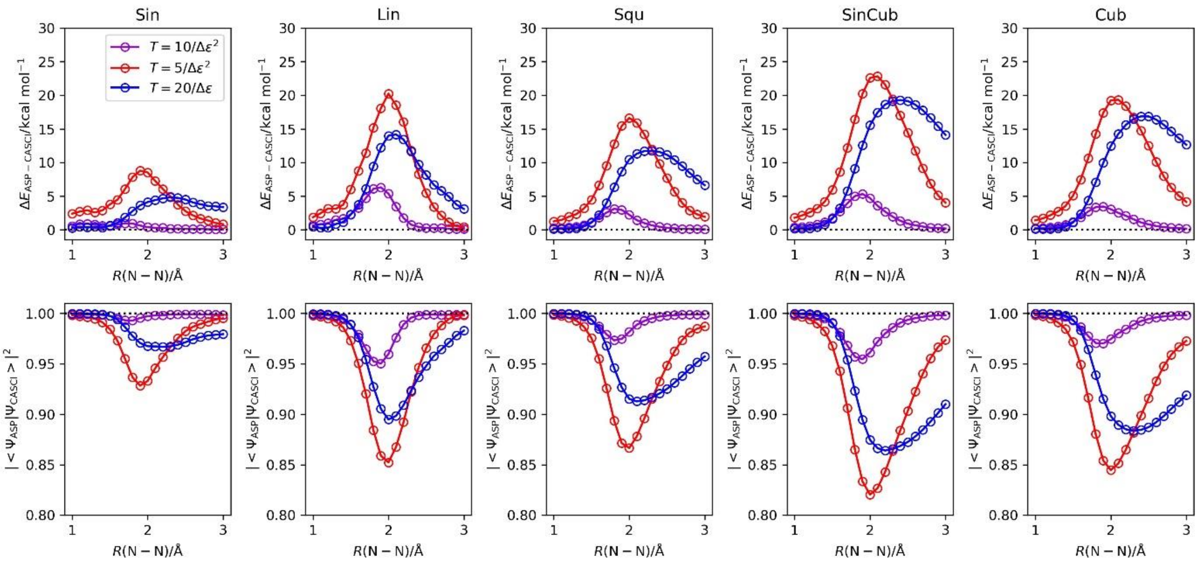

Fig. S2 Scheduling function dependence on the ASP wave functions with different evolution time length determination strategies in N_2 molecule. Top: The energy deviations from the CASCI values. Bottom: The square overlaps with the CASCI wave functions.

Supplementary Note 3

Scheduling function dependence on the numerical simulation of ASP with the $|\Psi_{BS}\rangle$ as the starting wave function in N_2 molecule at R(N–N) = 3.0 Å.

The results of the numerical quantum circuit simulation of ASP starting from the six-spin $|\Psi_{BS}\rangle$ (the wave function in Eq. 4 in the main text) in conjunction with *c* = 0.5, the evolution time length *T* = 10–100 of N_2 molecule at R(N–N) = 3.0 Å with the linear, sinusoidal, sinusoidal cubic, and cubic scheduling functions are summarized in Fig. S3–S6. The results obtained by using the square scheduling function are given in Fig. 6 in the main text.

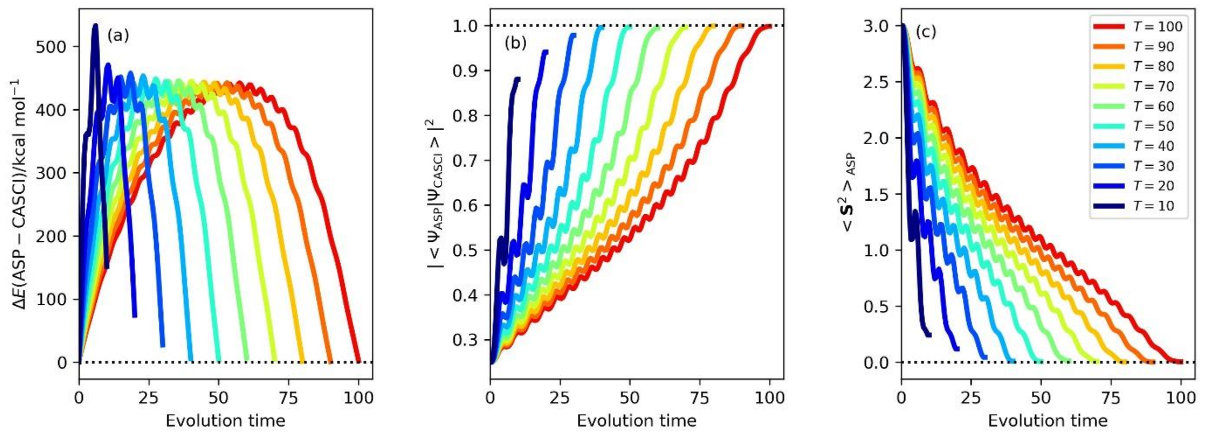

Fig. S3 Results of the numerical simulation of ASP with the $|\Psi_{BS}\rangle$ as the starting wave function and linear scheduling function in N_2 molecule at R(N–N) = 3.0 Å. (a) The energy deviations from the CASCI values. (b) The square overlaps with the CASCI wave functions. (c) The $\langle \mathbf{S}^2 \rangle$ values.

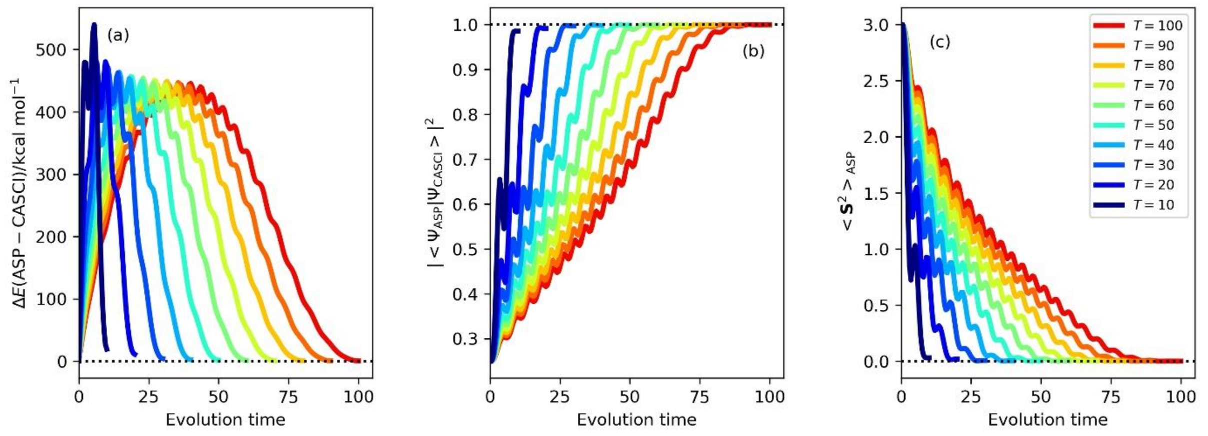

Fig. S4 Results of the numerical simulation of ASP with the $|\Psi_{BS}\rangle$ as the starting wave function and sinusoidal scheduling function in N_2 molecule at R(N–N) = 3.0 Å. (a) The energy deviations from the CASCI values. (b) The square overlaps with the CASCI wave functions. (c) The $\langle \mathbf{S}^2 \rangle$ values.

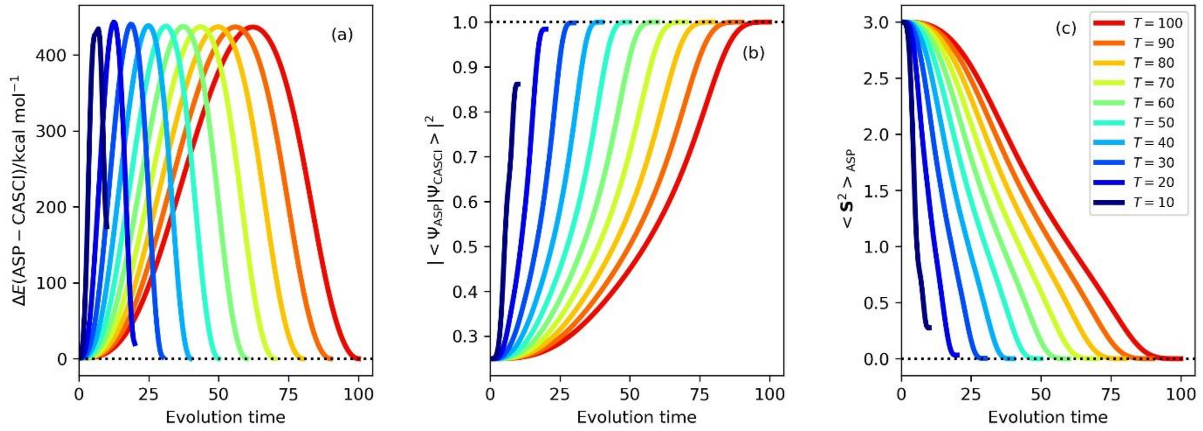

Fig. S5 Results of the numerical simulation of ASP with the $|\Psi_{BS}\rangle$ as the starting wave function and sinusoidal cubic scheduling function in N_2 molecule at R(N–N) = 3.0 Å. (a) The energy deviations from the CASCI values. (b) The square overlaps with the CASCI wave functions. (c) The $\langle \mathbf{S}^2 \rangle$ values.

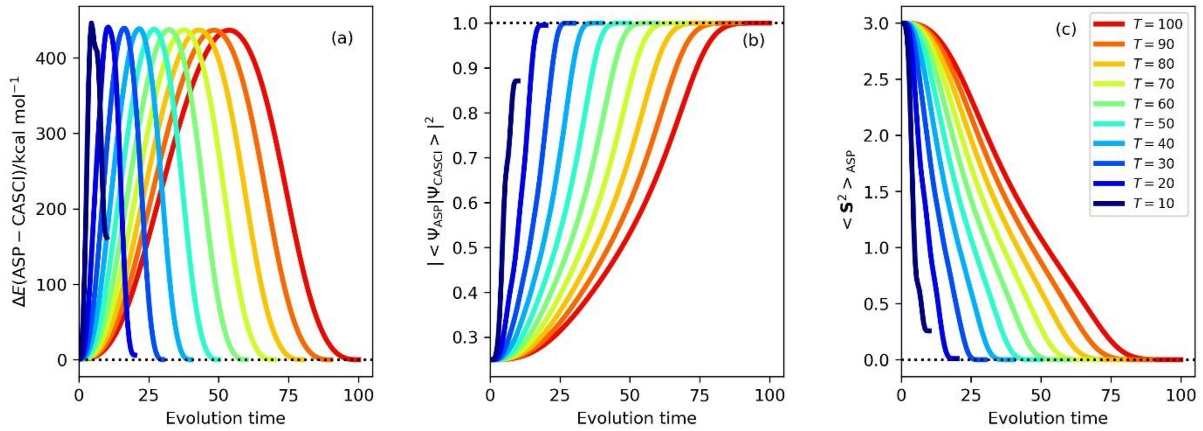

Fig. S6 Results of the **n**umerical simulation of ASP with the $|\Psi_{BS}\rangle$ as the starting wave function and cubic scheduling function in N_2 molecule at R(N–N) = 3.0 Å. (a) The energy deviations from the CASCI values. (b) The square overlaps with the CASCI wave functions. (c) The $\langle \mathbf{S}^2 \rangle$ values.

Supplementary Note 4

Numerical ASP simulations of N_2 molecule using the 6-31G* and 6-311G* basis sets with (6e,6o), (10e,8o), (6e,8o), and (10e,10o) active spaces.

The numerical quantum circuit simulations of ASP of N_2 molecule described in the main text are based on the (6e,6o) active space in conjunction with the STO-3G basis set. To disclose further insight of the convergence behavior of ASP and starting wave function selection based on the diradical character, we have carried out additional numerical simulations of ASP of N_2 molecule using 6-31G* and 6-311G* basis sets with (6e,6o), (10e,8o), (6e,8o), and (10e,10o) active spaces. We choose these active spaces based on the occupation number of natural orbitals constructed from the BS-UHF wave functions. The first 15 natural orbitals having larger occupation numbers generated from the BS-UHF/6-311G* wave function of N_2 at R(N–N) = 2.0 Å and corresponding occupation numbers are summarized in Fig. S7, as an example. From the occupation number of natural orbitals, we can select molecular orbitals which are important for the ground state wave function. The (6e,6o) active space consisting of valence σ/σ^* and π/π^* orbitals (MO indices 5–10) can be the minimal active space to qualitatively describe the triple bond dissociation of N_2 molecule. In addition, two occupied orbitals of the 2s character of N atoms (MO indices 3 and 4) and two virtual orbitals with 3s character of N atoms (MO indices 11 and 12) have the occupation numbers deviated more than 0.001 from 2 and 0, respectively. Thus, we decided to examine four types of active spaces: (6e,6o) active space containing orbitals 5–10, (10e,6o) active space consisting of orbitals 3–10, (6e,10o) active space with orbitals 5–12, and (10e,10o) active space with orbitals 3–12. After the localization of natural orbitals by mixing orbitals (7, 8), and (6, 9), we performed the ASP simulations starting from $|\Psi_{BS2}\rangle$ using the square scheduling function and setting the evolution time length T = 50. The ASP simulations with $|\Psi_{BS3}\rangle$ were carried out using the same computational conditions, after additional orbital localization procedure of orbitals (5, 10). In the ASP simulations with the $|\Psi_{HF}\rangle$ reference, using the RHF canonical orbitals directly is not plausible, because the RHF canonical orbitals are ordered by the orbital energy, rather than importance of the orbital in the ground state wave function. Using the natural orbitals from the BS-UHF wave function is one possible solution, but this approach is not applicable when the BS-UHF calculation converges to the RHF solution. In this work, we constructed the natural orbitals from the CISD calculations, and use them for the ASP starting from the HF configurations. We manually selected the natural orbitals which have the similar nature as the active orbitals used in the ASP with $|\Psi_{BS}\rangle$, so that the active spaces become consistent between the ASP starting from $|\Psi_{HF}\rangle$ and $|\Psi_{BS}\rangle$.

Results of the numerical simulations are summarized in Fig. S8–S15. By using the 6-31G* and 6-311G* basis sets, the ASP starting with $|\Psi_{BS2}\rangle$ gave somewhat poor results compared with those obtained from the STO-3G basis set (see Fig. 8 in the main text for comparison), but the tendency is the same among the STO-3G, 6-31G*, and 6-311G* basis sets.

MO index	1	2	3	4	5
MO plot	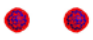	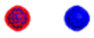	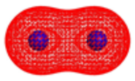	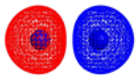	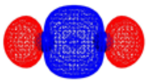
Occupation number	1.999997	1.999997	1.998561	1.996752	1.453404
	6	7	8	9	10
	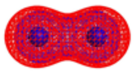	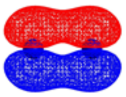	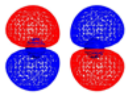	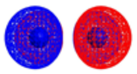	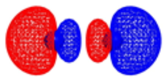
	1.139726	1.139726	0.860274	0.860274	0.546596
	11	12	13	14	15
	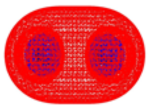	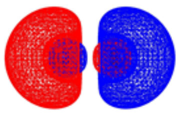	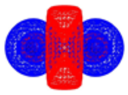	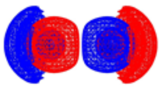	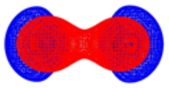
	0.003248	0.001439	0.000003	0.000003	0.000000

Fig. S7 Plots and occupation numbers of the first 15 natural orbitals having the largest occupation numbers of N_2 molecule at the geometry R(H–H) = 2.0 Å, constructed from the BS-UHF/6-311G* wave function.

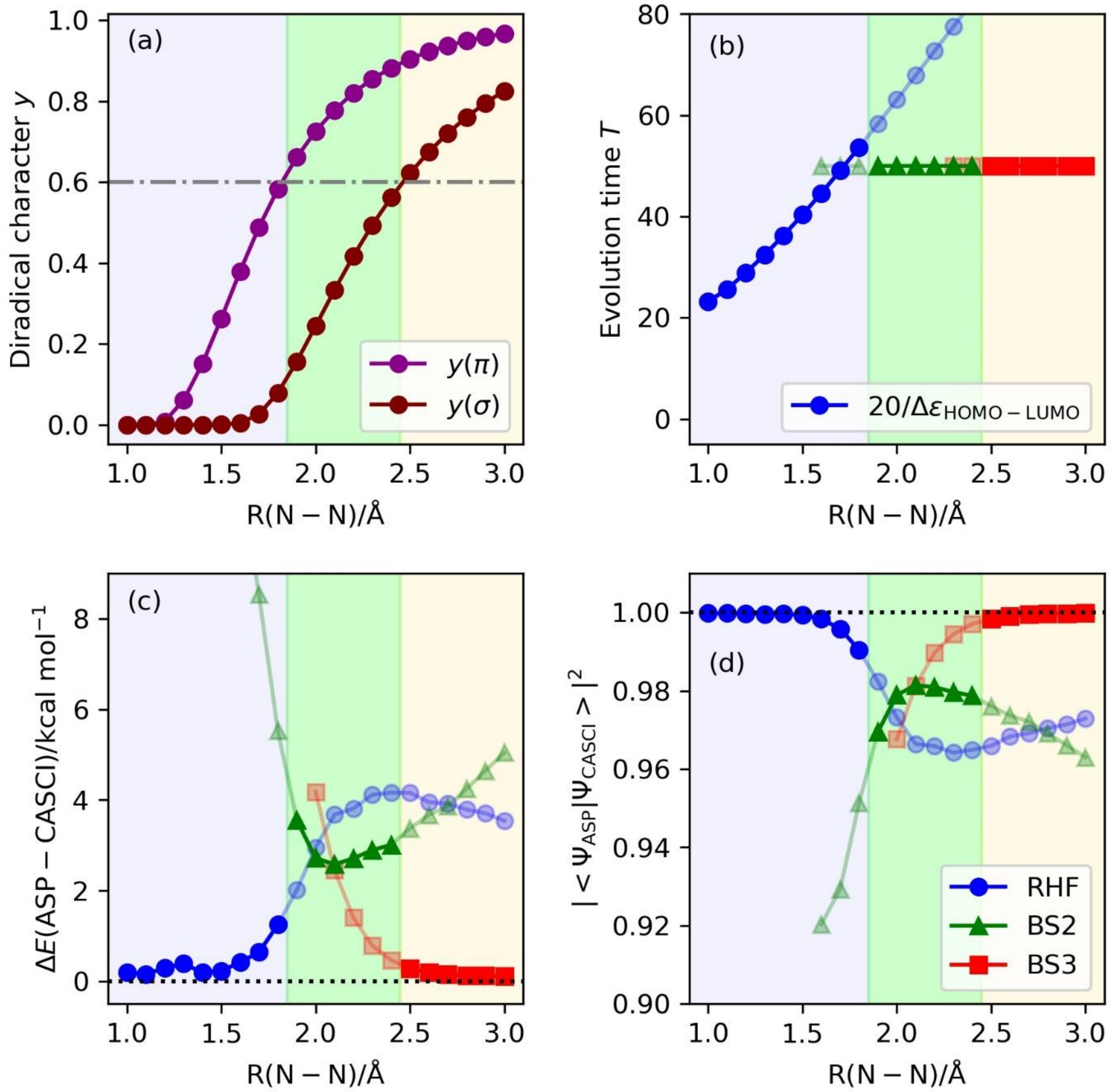

Fig. S8 Results of the numerical simulation of ASP for the potential energy curve of N_2 molecule using the (6e,6o) active space and 6-31G* basis set. Diradical character is used as the indicator for selecting the initial wave function. Background colors specify the regions of the initial wave function recommended from the criterion based on the diradical character y > 0.6. Sinusoidal and square functions were adopted as the scheduling functions with the $|\Psi_{HF}\rangle$ and $|\Psi_{BS}\rangle$, respectively, as the starting wave function. (a) Diradical characters calculated using Eq. 6 in the main text. (b) Evolution time length. (c) The energy differences from the CASCI values. (d) The square overlaps with the CASCI wave functions.

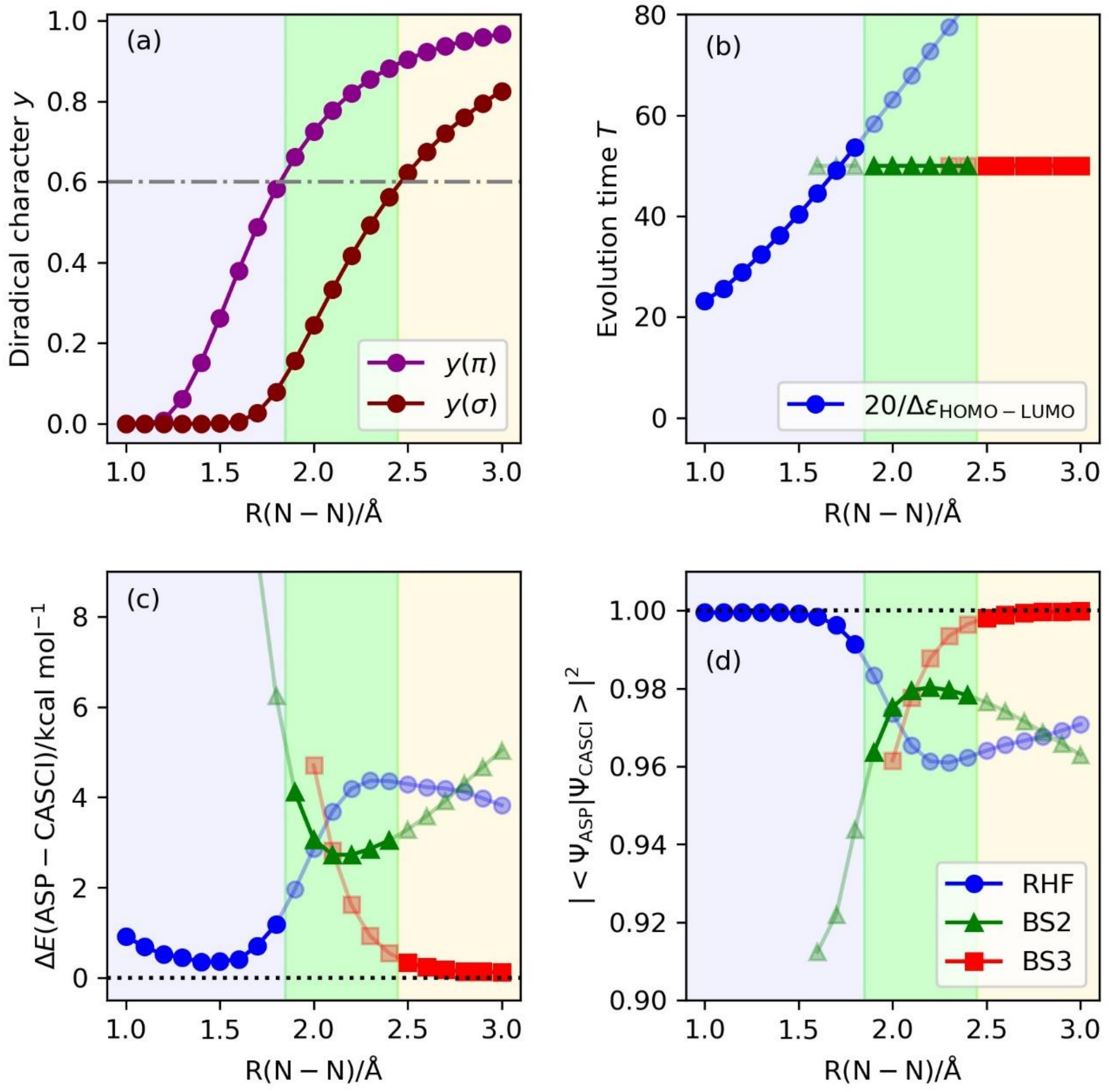

Fig. S9 Results of the numerical simulation of ASP for the potential energy curve of N_2 molecule using the (10e,8o) active space and 6-31G* basis set. Diradical character is used as the indicator for selecting the initial wave function. Background colors specify the regions of the initial wave function recommended from the criterion based on the diradical character $y > 0.6$. Sinusoidal and square functions were adopted as the scheduling functions with the $|\Psi_{HF}\rangle$ and $|\Psi_{BS}\rangle$, respectively, as the starting wave function. (a) Diradical characters calculated using Eq. 6 in the main text. (b) Evolution time length. (c) The energy differences from the CASCI values. (d) The square overlaps with the CASCI wave functions.

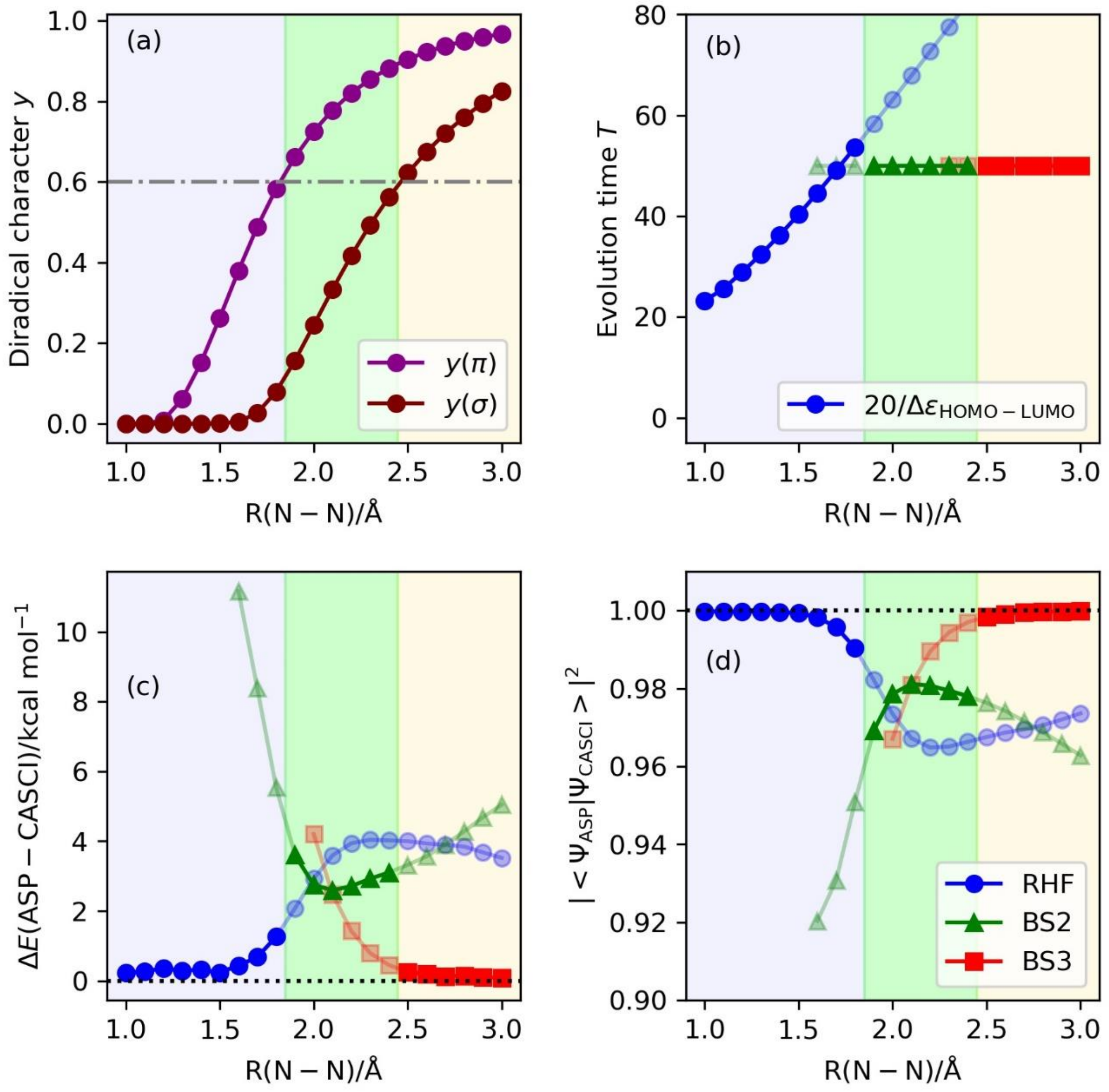

Fig. S10 Results of the numerical simulation of ASP for the potential energy curve of N_2 molecule using the (6e,8o) active space and 6-31G* basis set. Diradical character is used as the indicator for selecting the initial wave function. Background colors specify the regions of the initial wave function recommended from the criterion based on the diradical character y > 0.6. Sinusoidal and square functions were adopted as the scheduling functions with the $|\Psi_{HF}\rangle$ and $|\Psi_{BS}\rangle$, respectively, as the starting wave function. (a) Diradical characters calculated using Eq. 6 in the main text. (b) Evolution time length. (c) The energy differences from the CASCI values. (d) The square overlaps with the CASCI wave functions.

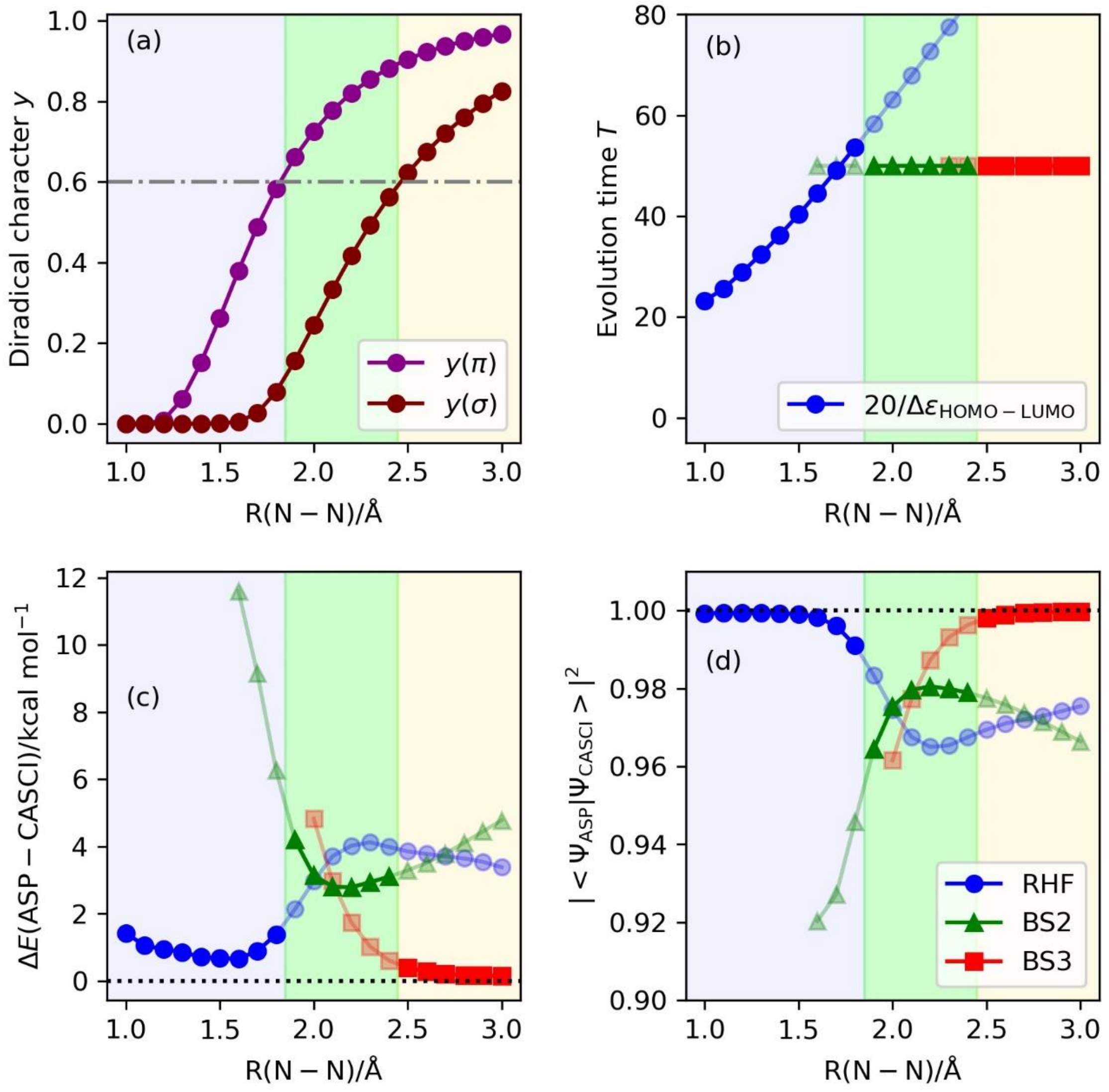

Fig. S11 Results of the numerical simulation of ASP for the potential energy curve of N_2 molecule using the (10e,10o) active space and 6-31G* basis set. Diradical character is used as the indicator for selecting the initial wave function. Background colors specify the regions of the initial wave function recommended from the criterion based on the diradical character $y > 0.6$. Sinusoidal and square functions were adopted as the scheduling functions with the $|\Psi_{HF}\rangle$ and $|\Psi_{BS}\rangle$, respectively, as the starting wave function. (a) Diradical characters calculated using Eq. 6 in the main text. (b) Evolution time length. (c) The energy differences from the CASCI values. (d) The square overlaps with the CASCI wave functions.

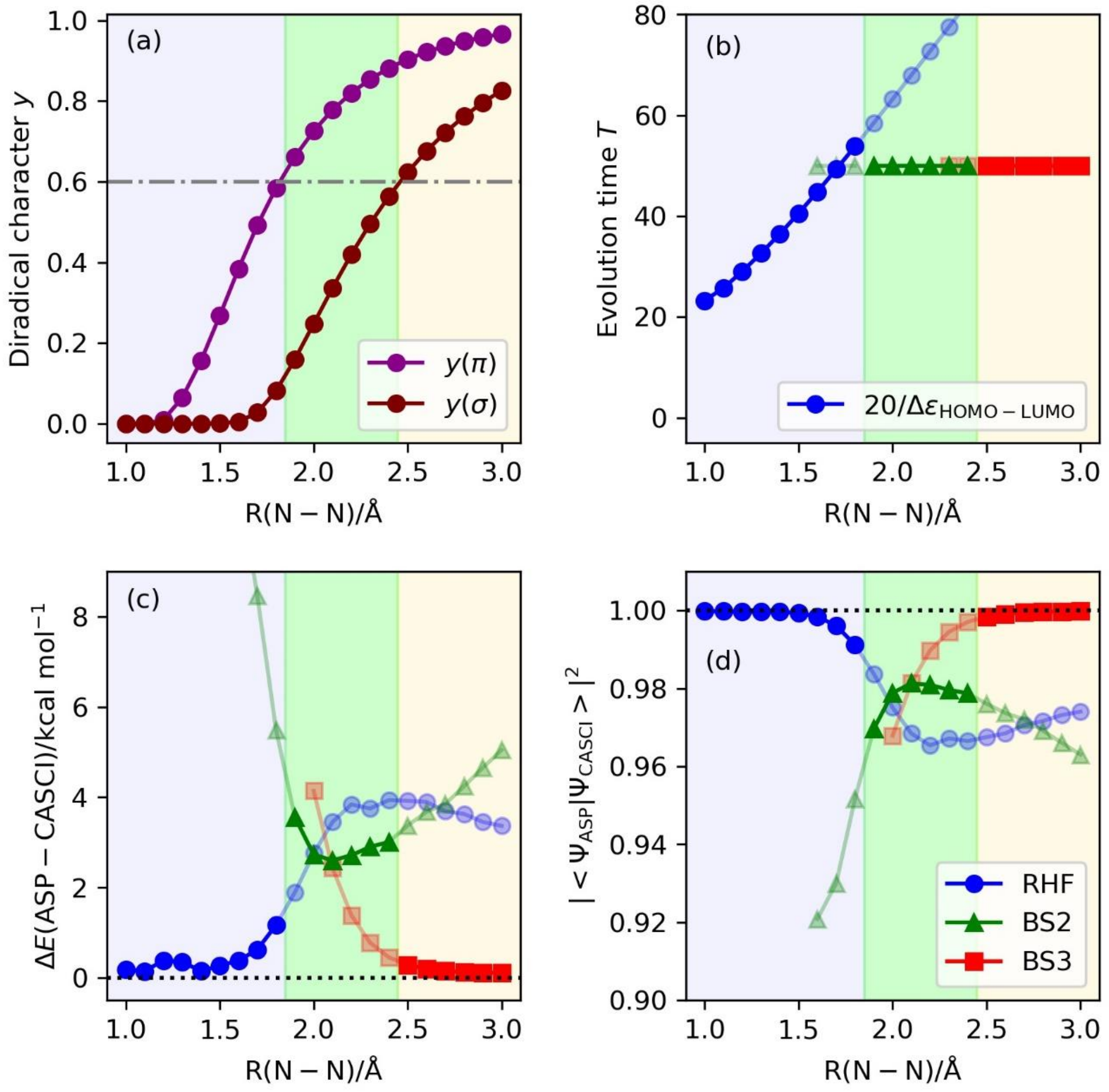

Fig. S12 Results of the numerical simulation of ASP for the potential energy curve of N_2 molecule using the (6e,6o) active space and 6-311G* basis set. Diradical character is used as the indicator for selecting the initial wave function. Background colors specify the regions of the initial wave function recommended from the criterion based on the diradical character $y > 0.6$. Sinusoidal and square functions were adopted as the scheduling functions with the $|\Psi_{HF}\rangle$ and $|\Psi_{BS}\rangle$, respectively, as the starting wave function. (a) Diradical characters calculated using Eq. 6 in the main text. (b) Evolution time length. (c) The energy differences from the CASCI values. (d) The square overlaps with the CASCI wave functions.

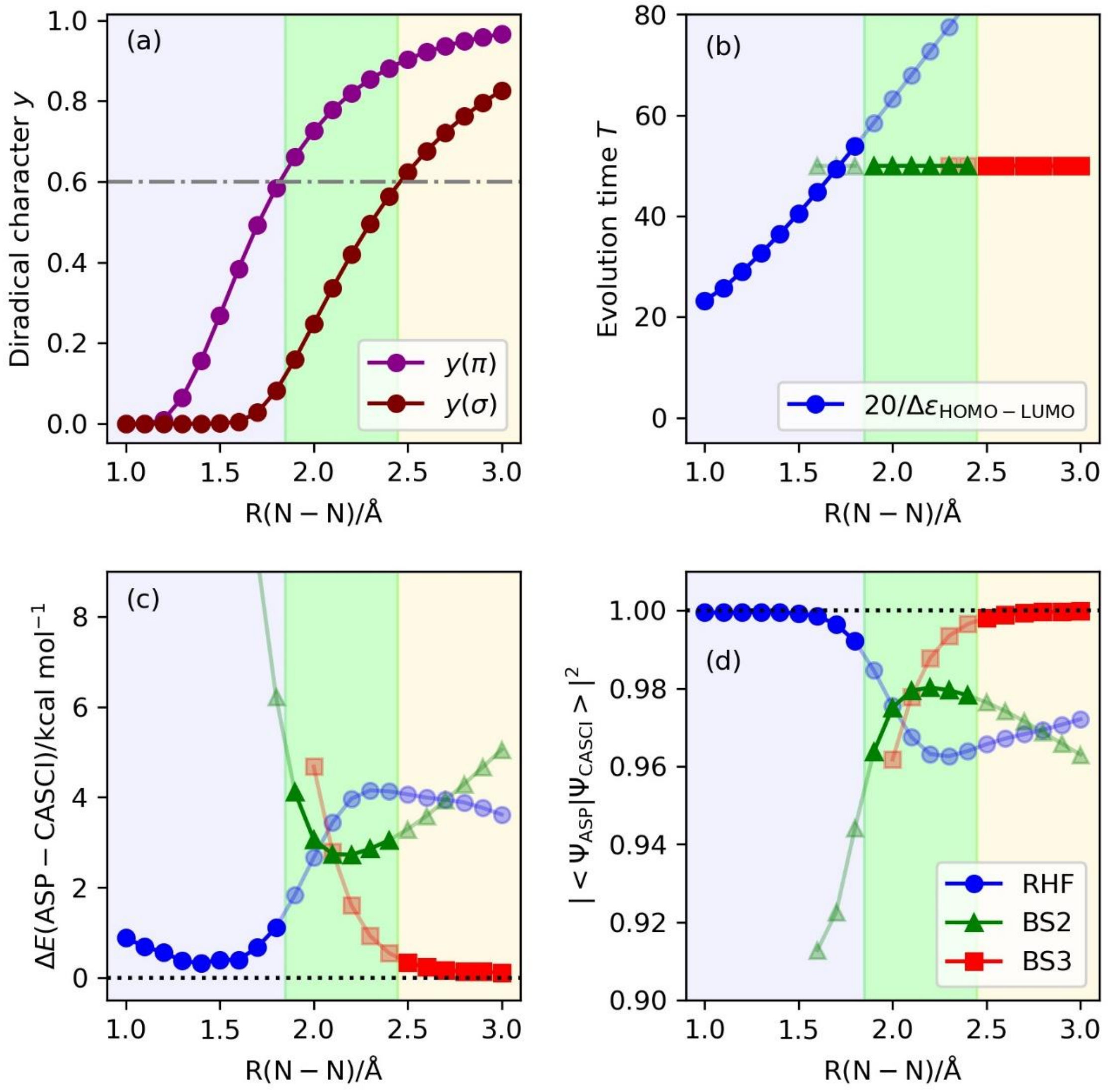

Fig. S13 Results of the numerical simulation of ASP for the potential energy curve of N_2 molecule using the (10e,8o) active space and 6-311G* basis set. Diradical character is used as the indicator for selecting the initial wave function. Background colors specify the regions of the initial wave function recommended from the criterion based on the diradical character y > 0.6. Sinusoidal and square functions were adopted as the scheduling functions with the $|\Psi_{HF}\rangle$ and $|\Psi_{BS}\rangle$, respectively, as the starting wave function. (a) Diradical characters calculated using Eq. 6 in the main text. (b) Evolution time length. (c) The energy differences from the CASCI values. (d) The square overlaps with the CASCI wave functions.

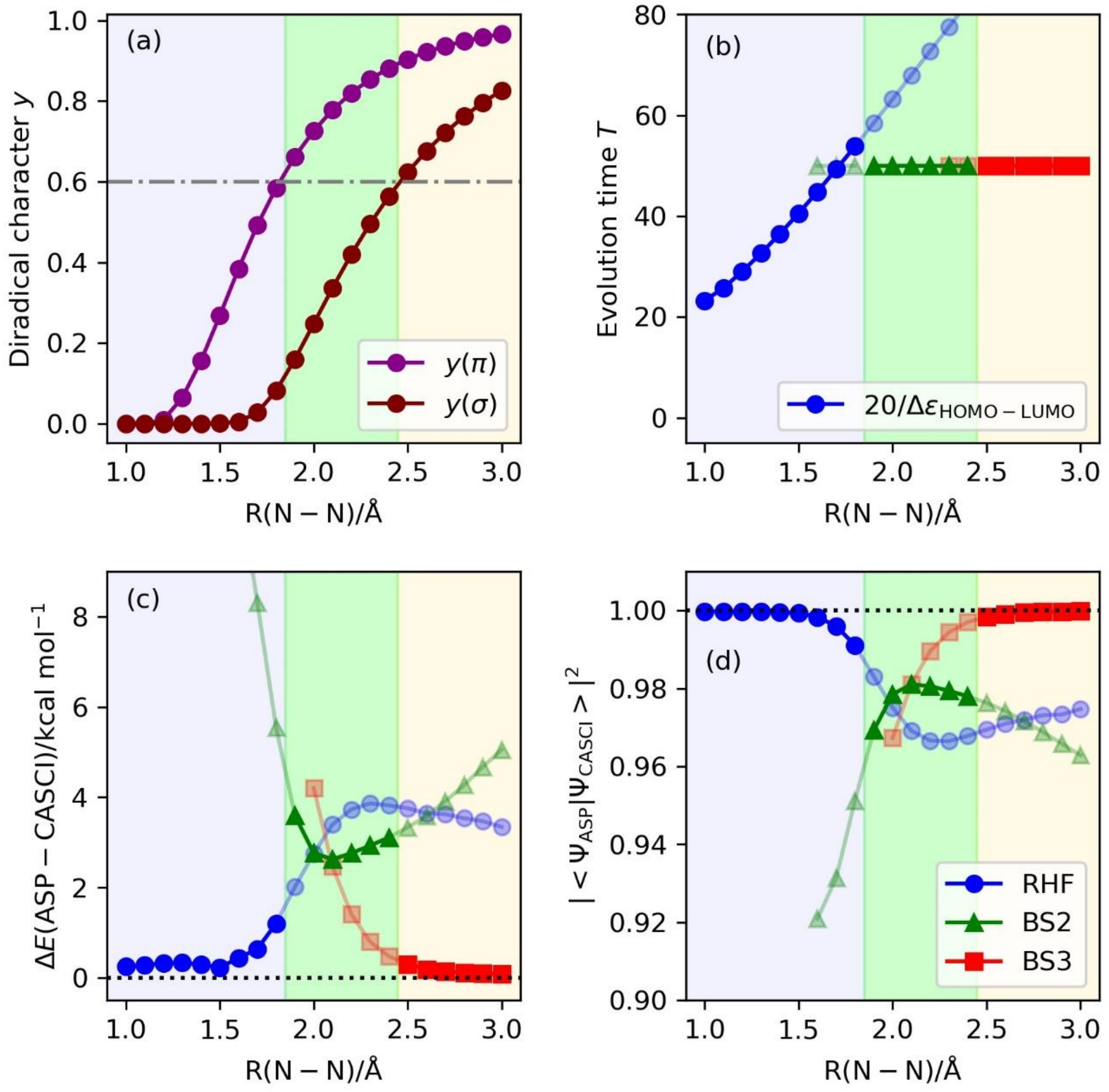

Fig. S14 Results of the numerical simulation of ASP for the potential energy curve of N_2 molecule using the (6e,8o) active space and 6-311G* basis set. Diradical character is used as the indicator for selecting the initial wave function. Background colors specify the regions of the initial wave function recommended from the criterion based on the diradical character y > 0.6. Sinusoidal and square functions were adopted as the scheduling functions with the $|\Psi_{HF}\rangle$ and $|\Psi_{BS}\rangle$, respectively, as the starting wave function. (a) Diradical characters calculated using Eq. 6 in the main text. (b) Evolution time length. (c) The energy differences from the CASCI values. (d) The square overlaps with the CASCI wave functions.

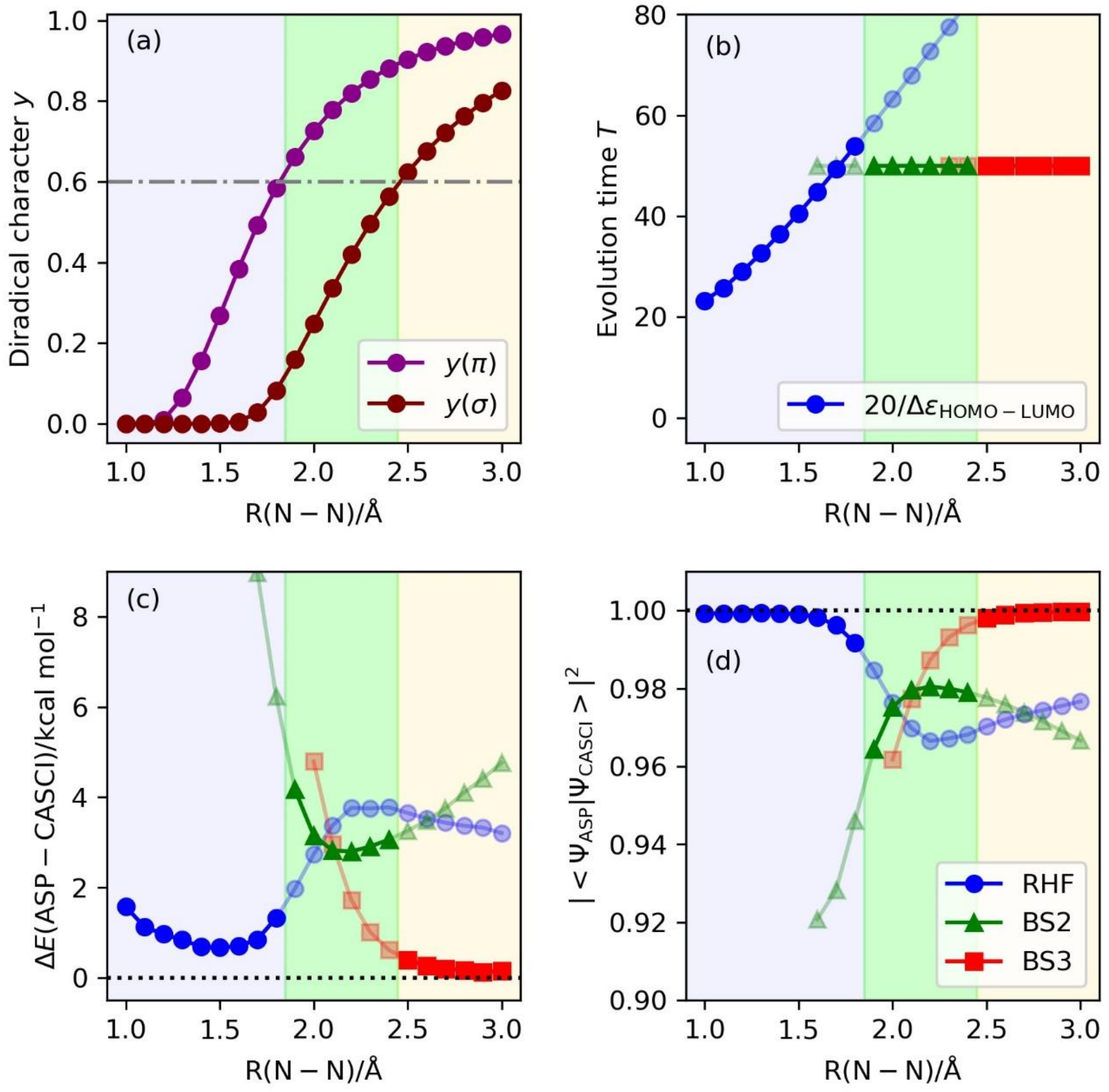

Fig. S15 Results of the numerical simulation of ASP for the potential energy curve of N_2 molecule using the (10e,10o) active space and 6-311G* basis set. Diradical character is used as the indicator for selecting the initial wave function. Background colors specify the regions of the initial wave function recommended from the criterion based on the diradical character y > 0.6. Sinusoidal and square functions were adopted as the scheduling functions with the $|\Psi_{HF}\rangle$ and $|\Psi_{BS}\rangle$, respectively, as the starting wave function. (a) Diradical characters calculated using Eq. 6 in the main text. (b) Evolution time length. (c) The energy differences from the CASCI values. (d) The square overlaps with the CASCI wave functions.

Supplementary Note 5

Results of the numerical simulation of ASP for the BeH_2 potential energy surface with longer evolution times.

To check the convergence behavior of ASP in the potential energy curve of BeH_2 molecule, we have executed numerical ASP simulations with longer evolution times for the geometries R(Be–H) between 2.0 to 3.0 Å. The results are summarized in Fig. S16 and S17 for $|\Psi_{HF}\rangle$ and $|\Psi_{BS}\rangle$, respectively, as the starting wave function. When $|\Psi_{HF}\rangle$ is adopted as the starting wave function in ASP, we can obtain $|\Psi_{ASP}\rangle$ with $|\langle\Psi_{ASP}|\Psi_{CASCI}\rangle|^2 > 0.999$ at $T = 200$, and no further improvement was acquired for $T > 200$. The ASP with $|\Psi_{BS}\rangle$ gave $|\langle\Psi_{ASP}|\Psi_{CASCI}\rangle|^2 > 0.998$ at $T = 100$. Acquiring very accurate wave function by using ASP is more difficult for the systems with intermediate open shell characters (diradical character $y \sim 0.5$) than the systems with $y \sim 0.0$ (dominant closed shell singlet configurations) and $y \sim 1.0$ (singlet diradicaloid), but our numerical simulations exemplify that we can improve the quality of $|\Psi_{ASP}\rangle$ by increasing the evolution time.

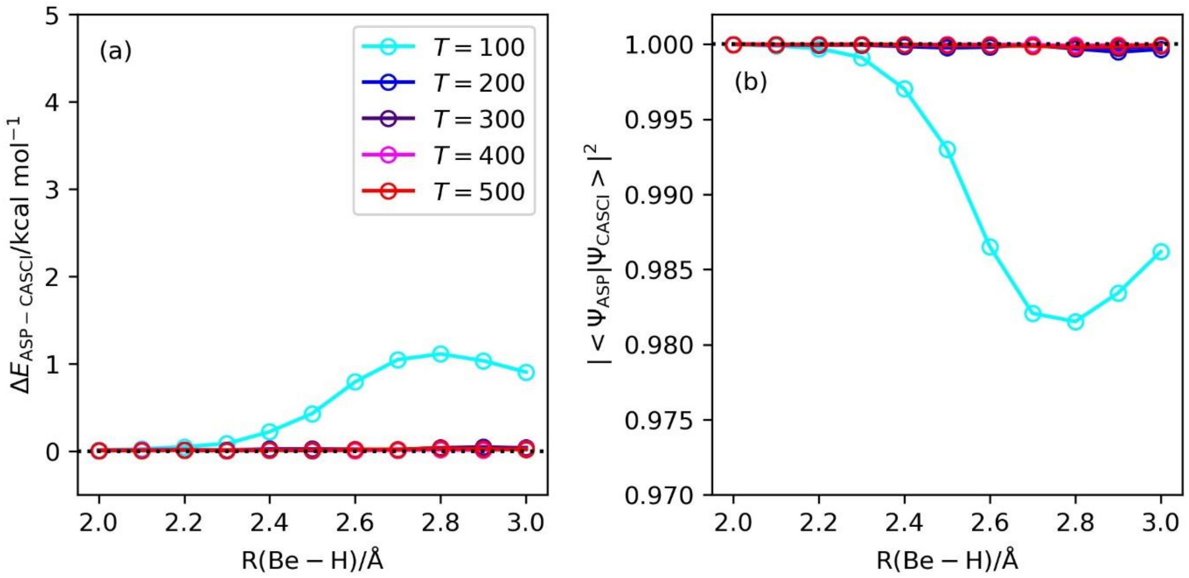

Fig. S16 Results of the numerical simulation of ASP with the $|\Psi_{HF}\rangle$ as the starting wave function in the potential energy curve of BeH_2 molecule with different evolution time length *T*. (a) The energy deviations from the CASCI values. (b) The square overlaps with the CASCI wave functions.

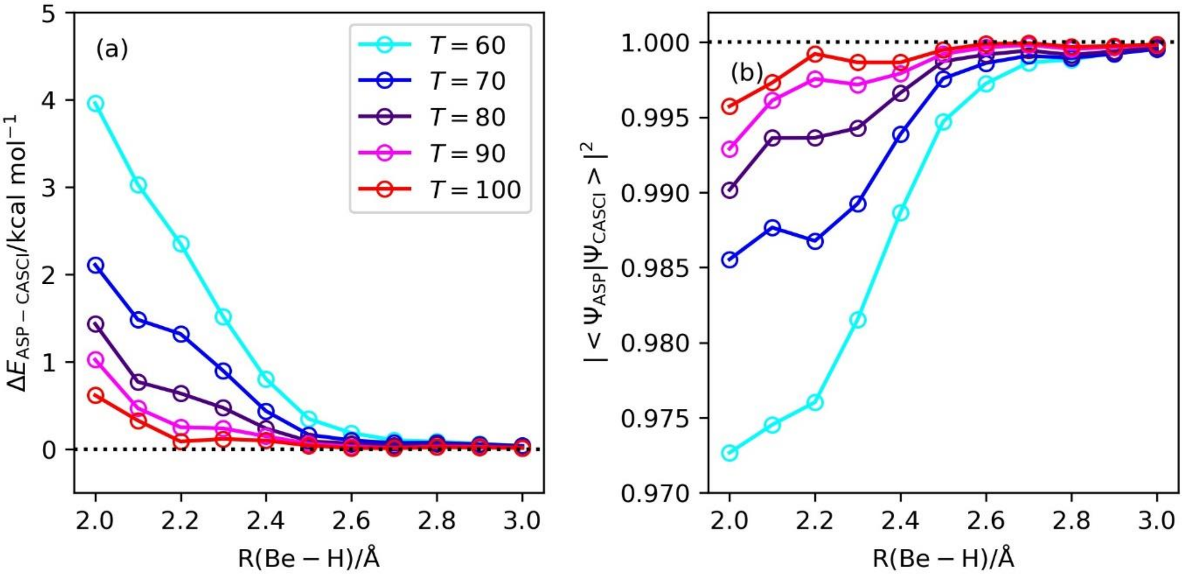

Fig. S17 Results of the numerical simulation of ASP with the $|\Psi_{BS}\rangle$ as the starting wave function in the potential energy curve of BeH_2 molecule with different evolution time length T. (a) The energy deviations from the CASCI values. (b) The square overlaps with the CASCI wave functions.

Supplementary Note 6

Results of the numerical simulation of ASP with different evolution time length determination strategies in BeH_2.

Numerical simulation results of ASP by using the sinusoidal, linear, square, sinusoidal cubic, and cubic scheduling functions for ASP with the $|\Psi_{HF}\rangle$, in conjunction with the evolution time length T = $20/\Delta\varepsilon$ are summarized in Fig. S18. Among the five scheduling functions being examined, the sinusoidal function gave the smallest $\Delta E_{ASP-CASCI}$ and the largest $|\langle\Psi_{ASP}|\Psi_{CASCI}\rangle|^2$ values, at the intermediate bond dissociation region with strong electron correlations (R(Be–H) = 2–3 Å).

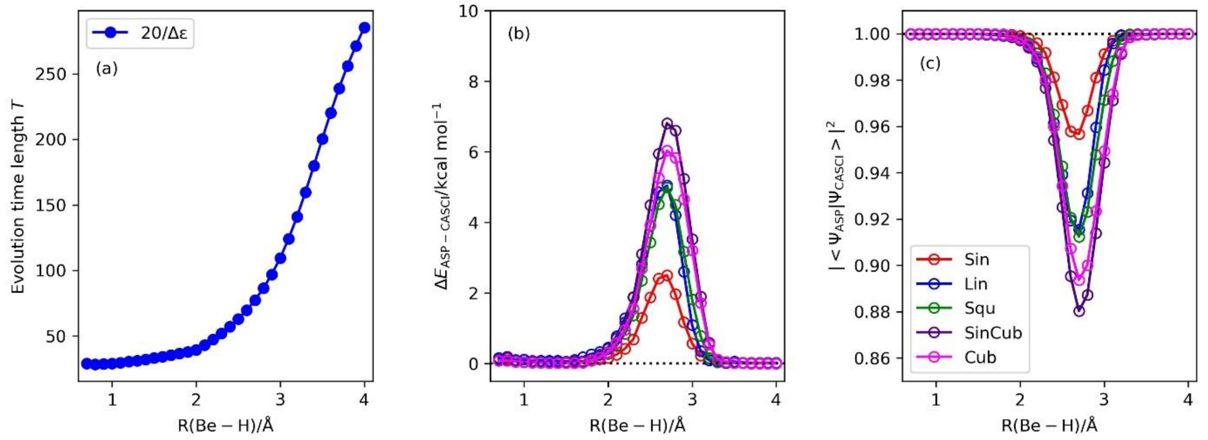

Fig. S18 Results of the numerical simulation of ASP with different evolution time length determination strategies in BeH_2. (a) Evolution time length. (b) The energy deviations from the CASCI values. (c) The square overlaps with the CASCI wave functions.

Supplementary Note 7

Numerical ASP simulations around the transition states of the Be + H_2 → BeH_2 quasi-reaction pathway.

As discussed in the main text, the ASP simulations using $|\Psi_{HF}\rangle$ as the starting wave function converge very slowly at point E of the Be + H_2 → BeH_2 quasi-reaction pathway. To investigate the convergence behavior of ASP around the transition structure in more detail, we carried out the ASP simulations at four additional geometries between points D and F specified as Z26–Z29, whose cartesian coordinates are given in Table S2.

Table S2. Cartesian coordinates of H atoms for the point being calculated. The Be atom is located at the origin of coordinates.

Point	X	Y	Z
D	0.000	±1.390	2.500
Z26	0.000	±1.344	2.600
Z27	0.000	±1.298	2.700
E	0.000	±1.275	2.750
Z28	0.000	±1.252	2.800
Z29	0.000	±1.206	2.900
F	0.000	±1.160	3.000

The square overlaps between the wave functions obtained from ASP and the CASCI calculations are 0.9809, 0.9125, 0.8525, and 0.9702 for Z26, Z27, Z28, and Z29, respectively, when the $|\Psi_{HF}\rangle$ is used as the starting wave function in ASP. The square overlap values decreased as the geometry approaches the transition structure. By using the $|\Psi_{BS}\rangle$ as the stating wave functions, the square overlaps $|\langle\Psi_{ASP}|\Psi_{CASCI}\rangle|^2$ were calculated to be 0.9993, 0.9993, 0.9992, and 0.9988 for Z26, Z27, Z28, and Z29, respectively. Again, ASP with $|\Psi_{BS}\rangle$ gave more accurate wave function than that with $|\Psi_{HF}\rangle$.

To disclose the reason why the ASP with $|\Psi_{HF}\rangle$ converges so slowly around point E, we calculated the S_1–S_0 energy gap of instantaneous Hamiltonian at point E. The results are plotted in Fig. S19. Clearly, the S_1 and S_0 states become almost gapless around $s(x)$ = 0.9. To study the active space dependence on the convergence behavior, we have executed the 16, 14, and 12 qubit ASP simulations by removing the highest virtual orbitals one by one. The square overlap values $|\langle\Psi_{ASP}|\Psi_{CASCI}\rangle|^2$ were calculated to be 0.8464, 0.853, and 0.9751 for 16, 14, and 12 qubit simulations, respectively. These results exemplify that selecting appropriate active orbitals is very important to obtain accurate wave functions from ASP. In this context, natural orbital-based wave function expansion is expected to be crucial, especially when the extended basis set is employed and the wave function has many virtual orbitals. In the next section we examined the ASP simulations of Be + H_2 → BeH_2 reaction systems using CISD/6-31G* natural orbitals.

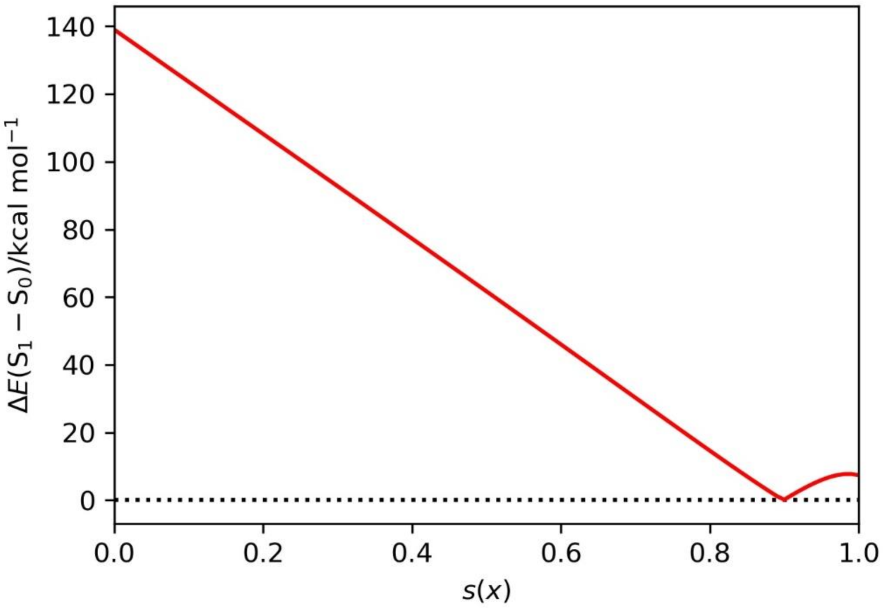

Fig. S19 The S_1–S_0 energy gap of the instantaneous Hamiltonian at point E in the Be + H_2 → BeH_2 quasi-reaction pathway.

Supplementary Note 8

Numerical ASP simulations of the Be + H_2 → BeH_2 quasi-reaction pathway by using the 6-31G* basis set.

The results of numerical simulations discussed in the previous section suggest importance of the active orbital selection based on the occupation number of natural orbitals, especially when the basis set larger than the minimal basis is employed. To further investigate the effect of the size of active space and basis set on the convergence behavior of ASP, we have carried out ASP numerical simulations of the Be + H_2 → BeH_2 quasi-reaction pathway using (4e,8o) active space in conjunction with 6-31G* basis set. This active space was selected based on the occupation number of the natural orbitals constructed from the CISD/6-31G* wave function. The CISD/6-31G* natural orbitals and corresponding occupation numbers at point E are summarized in Fig. S20. Note that we have adopted the frozen-core approximation in the CISD calculations. We selected the natural orbitals with the occupation number deviated 0.001 or larger from 2 (occupied) or 0 (unoccupied), for the active space at point E. The active spaces of other points were constructed manually, to make the active spaces of all points being consistent. The results of quantum circuit simulations were summarized in Fig. S21. By appropriately selecting the active orbitals, we could obtain the $|\Psi_{ASP}\rangle$ having the square overlap with the CASCI wave function larger than 0.98 for all the point being studied.

MO index	1	2	3	4	5	6
MO plot	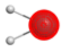	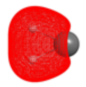	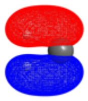	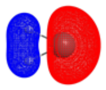	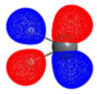	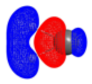
Occ. number	2.000000	1.943123	1.752062	0.217965	0.064370	0.007184
7	8	9	10	11	12	13
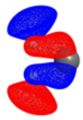	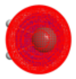	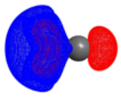	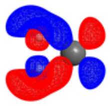	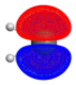	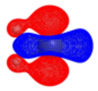	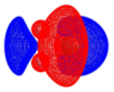
0.005193	0.003502	0.002859	0.000884	0.000816	0.000591	0.000499
14	15	16	17	18	19	
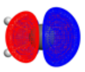	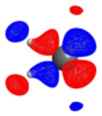	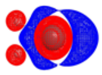	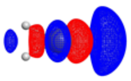	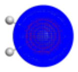	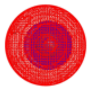	
0.000472	0.000243	0.000157	0.000062	0.000008	0.000008	

Fig. S20 Plots and occupation numbers of the natural orbitals constructed from CISD/6-31G* wave function at point E in the Be + H_2 → BeH_2 quasi-reaction pathway.

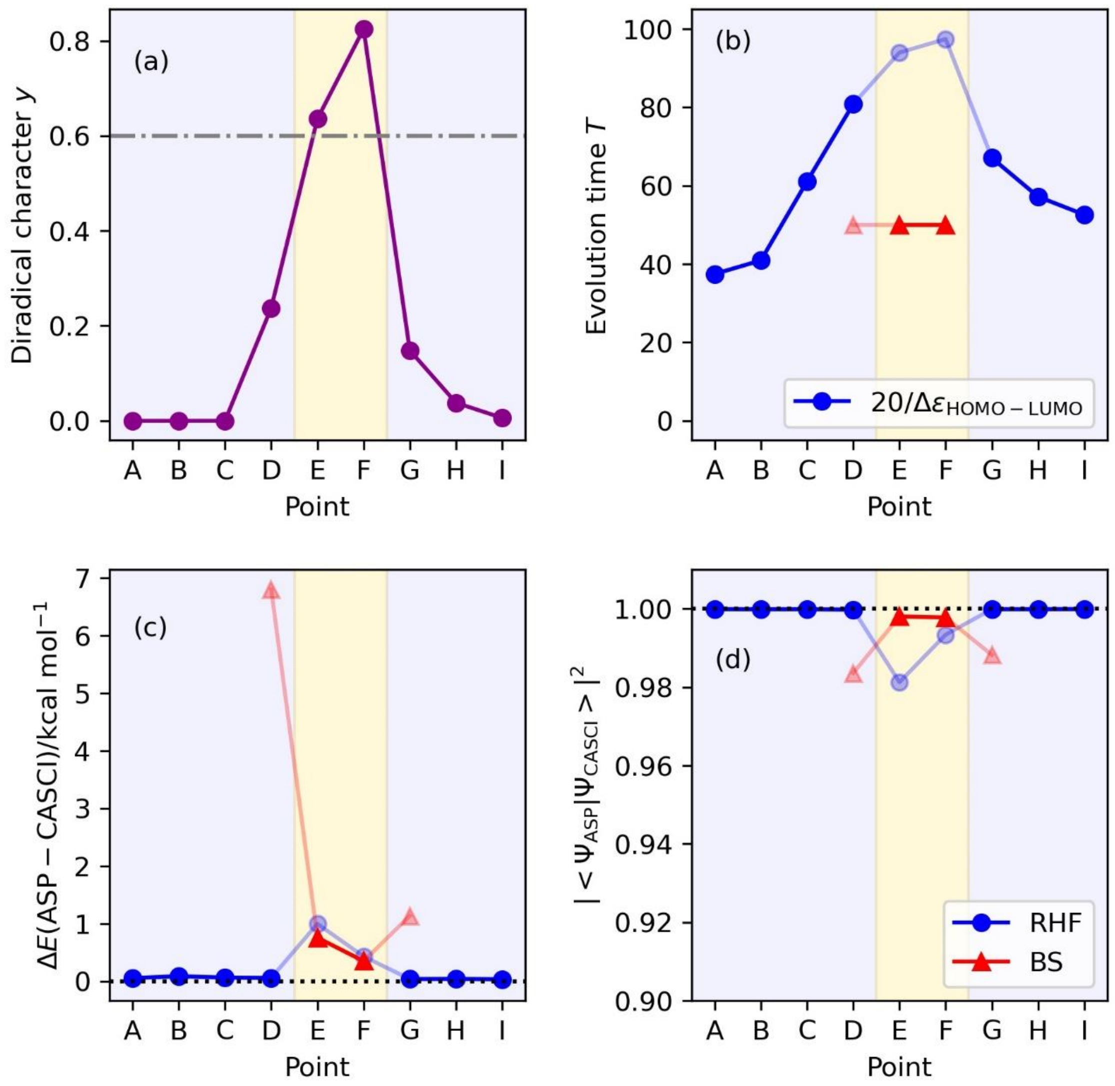

Fig. S21 Results of the numerical simulation of ASP of the C_{2v} quasi-reaction pathway of Be + H_2 → BeH_2, using the (4e,8o) active space and 6-31G* basis set. Diradical character is used as the indicator for selecting the initial wave function. Background colors specify the regions of the initial wave function recommended from the criterion based on the diradical character y > 0.6. Sinusoidal and square functions were adopted as the scheduling functions with the $|\Psi_{HF}\rangle$ and $|\Psi_{BS}\rangle$, respectively, as the starting wave function. (a) Diradical characters calculated using Eq. 6 in the main text. (b) Evolution time length. (c) The energy differences from the CASCI values. (d) The square overlaps with the CASCI wave functions.
